# Polyunsaturated fatty acid metabolism in the retinal pigment epithelium and its association with outer retinal disease

**DOI:** 10.1007/s00335-026-10239-y

**Published:** 2026-05-22

**Authors:** Naga Pradeep Rayana, Navdeep Gogna, Mark P. Krebs, Gayle B. Collin, Jürgen K. Naggert, Patsy M. Nishina

**Affiliations:** 1 The Jackson Laboratory, Bar Harbor, ME 04609 USA; 2 Department of Neuroscience, Graduate School of Biomedical Sciences, Tufts University, Boston, MA 02111 USA

**Keywords:** Retinal pigment epithelium, Photoreceptors, Polyunsaturated fatty acids metabolism, Endoplasmic reticulum, Lipid droplets, Peroxisomes, Mitochondria, Lipid homeostasis

## Abstract

Polyunsaturated fatty acids (PUFAs) are essential for photoreceptor (PR) development, structure and function, and their availability in the outer retina is predominantly mediated by the retinal pigment epithelium (RPE), a cellular monolayer juxtaposed to the PR outer segments. Because PRs lack enzymatic machinery to generate critical PUFA intermediates from dietary precursors, they rely on the RPE to coordinate multiple steps of PUFA uptake, metabolism and export. Consequently, disruption in any of these steps perturbs PR homeostasis and compromises overall retinal health. In this review, we summarize current evidence about the genes, proteins, molecular pathways, and pathological alterations that govern the regulation of PUFA metabolism in the RPE. Data from human genetic disorders, mouse models and in vitro studies is discussed to illustrate how disruption of PUFA pathways in the RPE leads to lipid imbalance and retinal degeneration, and to highlight how these findings illuminate molecular mechanisms underlying PUFA biology. We also identify critical gaps in knowledge and unresolved questions surrounding RPE-PR PUFA metabolism and propose that addressing these gaps will be essential for advancing therapeutic strategies for retinal disease.

## Introduction

Visual phototransduction—the process by which light is converted into neural signals by PR cells in the mammalian retina—relies heavily on the unique lipid composition of photoreceptor outer segment (POS) membranes, which are highly enriched in PUFAs. These fatty acids (FAs) play essential structural and functional roles in visual signaling (Swinkels and Baes 2023; Uauy et al. 2001). PUFAs are defined as FAs containing more than 12 carbon atoms and two or more double bonds within their hydrocarbon chain. Based on chain length, they are classified as long chain-PUFA (LC-PUFA; C20-C22) or very long chain-PUFA (VLC-PUFA; > C24 extending up to 36 carbons). PUFAs are further categorized according to the position of the first double bond from the methyl end, giving rise to omega-3(ω-3) and omega-6(ω-6) families.

Prominent ω-3 FAs, such as α-linolenic acid (ALA; 18:3n-3), eicosapentaenoic acid (EPA; 20:5n-3), and docosahexaenoic acid (DHA; 22:6n-3), as well as ω-6 FAs, such as linoleic acid (LA; 18:2n-6) and arachidonic acid (AA; 20:4n-6) (Mititelu et al. 2024) are described by a set of numbers separated by a colon. In this nomenclature, the first number represents the total number of carbon atoms in the chain, and the number following the colon indicates the number of double bonds (Recommendations 1977).

LC-PUFAs have broad physiological importance across multiple tissues (Janssen and Kiliaan 2014; Virk et al. 2024). Major LC-PUFAs like DHA are primarily obtained from the diet in esterified form and are transported in the circulation either associated with lipoproteins or as lysophospholipids. DHA is also produced inefficiently by endogenous synthesis through enzymatic conversion of dietary precursors like ALA (Calder 2015; Kris-Etherton et al. 2002). Owing to their multiple *cis* double bonds, LC-PUFAs adopt kinked conformations that prevent tight phospholipid packaging, thereby increasing membrane fluidity, curvature and permeability (Harayama and Shimizu 2020; Stillwell and Wassall 2003). These PUFA-mediated biophysical changes influence membrane bilayer organization and can modulate lipid microdomains, including cholesterol and sphingolipid-rich ordered phases, thereby affecting membrane protein distribution, receptor function and downstream signaling (Wassall and Stillwell 2009). Through incorporation into plasma, synaptic and organellar membranes, LC-PUFAs contribute to membrane remodeling, vesicle fusion and neurogenesis. Together, these roles make LC-PUFAs fundamental determinants of membrane architecture and function particularly in membrane systems that are dynamically and rapidly remodeled (Janssen and Kiliaan 2014; Salem et al. 2001).

VLC-PUFAs are particularly enriched in the phospholipids of photoreceptor membranes, where they are thought to serve specialized structural and functional roles (Aveldano and Sprecher 1987; Berdeaux et al. 2010). With their extended acyl chains and a high degree of planar double bonds, VLC-PUFAs influence membrane thickness, curvature, flexibility and lipid packing, helping to support specialized membrane domains (Aveldano 1987; Cheng et al. 2022). They have been proposed to facilitate transbilayer interactions, stabilize membrane components and modulate protein-lipid interactions (Aveldano and Sprecher 1987; Bennett et al. 2014; Yeboah et al. 2021). These biophysical properties likely underlie their roles in stabilizing specialized membrane domains and supporting tissue specific functions, such as the PR outer segment discs. While the specialized POS membranes are characterized by high levels of DHA-containing VLC-PUFAs, the RPE with more typical membrane requirements has only moderate levels of ω-3 and ω-6 LC-PUFAs and relatively low levels of VLC-PUFAs (Liu et al. 2010).

The retina is a major site of PUFA concentration with DHA comprising ~ 50% of the FAs in the membrane lipids of the PR outer segment (POS) discs (Fliesler and Anderson 1983; Stinson et al. 1991; Swinkels and Baes 2023). DHA is necessary for maintaining POS disc membrane fluidity, which is essential for optimizing membrane packing and mobility of phototransduction proteins, such as rhodopsin (Bazan 2006; Gawrisch et al. 2008; Litman and Mitchell 1996; Senapati et al. 2018). In addition to their critical role in visual function, PUFAs support retinal development by promoting the differentiation of PR progenitors and enhancing POS formation and opsin expression (Arai et al. 2017; Garelli et al. 2006; Politi et al. 2001; West et al. 2022). PUFAs are also precursors to a diverse array of bioactive oxidative products that regulate key cellular processes, generating mediators that promote PR survival, suppress inflammation, and support retinal homeostasis (Bazan 2009; Sacca et al. 2018; Zhang and Bazan 2010). Frequently, deficits in PUFA metabolism or dietary DHA deficiency lead to retinal structural defects and vision impairment (Bush et al. 1994; Jeffrey et al. 2002; Jeffrey and Neuringer 2009; Shindou et al. 2017; Swinkels et al. 2023).

The RPE is a cellular monolayer situated between the outer retina and the capillary bed of the choroid that controls the flux of molecules essential for vision, including PUFAs (Lewandowski et al. 2022b). Microvilli-like projections on the apical surface of the RPE engage the POS tips to promote retinal homeostasis (Kevany and Palczewski 2010; Strauss 2005; Young and Bok 1969). PRs continuously synthesize new POS discs, including the visual transduction machinery and membrane lipids. In a daily renewal cycle, ~ 10% of POS tips are shed by the PRs and phagocytosed by the RPE (LaVail 1976; Young 1978), necessitating a robust system for lipid turnover and recycling (Lewandowski et al. 2022b). Thus, the RPE acts both as a gateway between the systemic circulation and the retina, controlling the uptake of nutrients from and the removal of waste products to the systemic circulation, and as a recycling center that recovers and restores critical molecules, returning them to the retina for reuse in PR maintenance and the phototransduction process (Lakkaraju et al. 2020; Lewandowski et al. 2022b; O’Leary and Campbell 2023). As the synthesis of key PUFAs and their precursors in the retina is insufficient for the lifelong maintenance of PRs, the RPE has emerged as a tissue that is critical for regulating ocular PUFA metabolism (Bazan et al. 1992; Fu et al. 2021; Swinkels and Baes 2023).

**Fig. 1:**
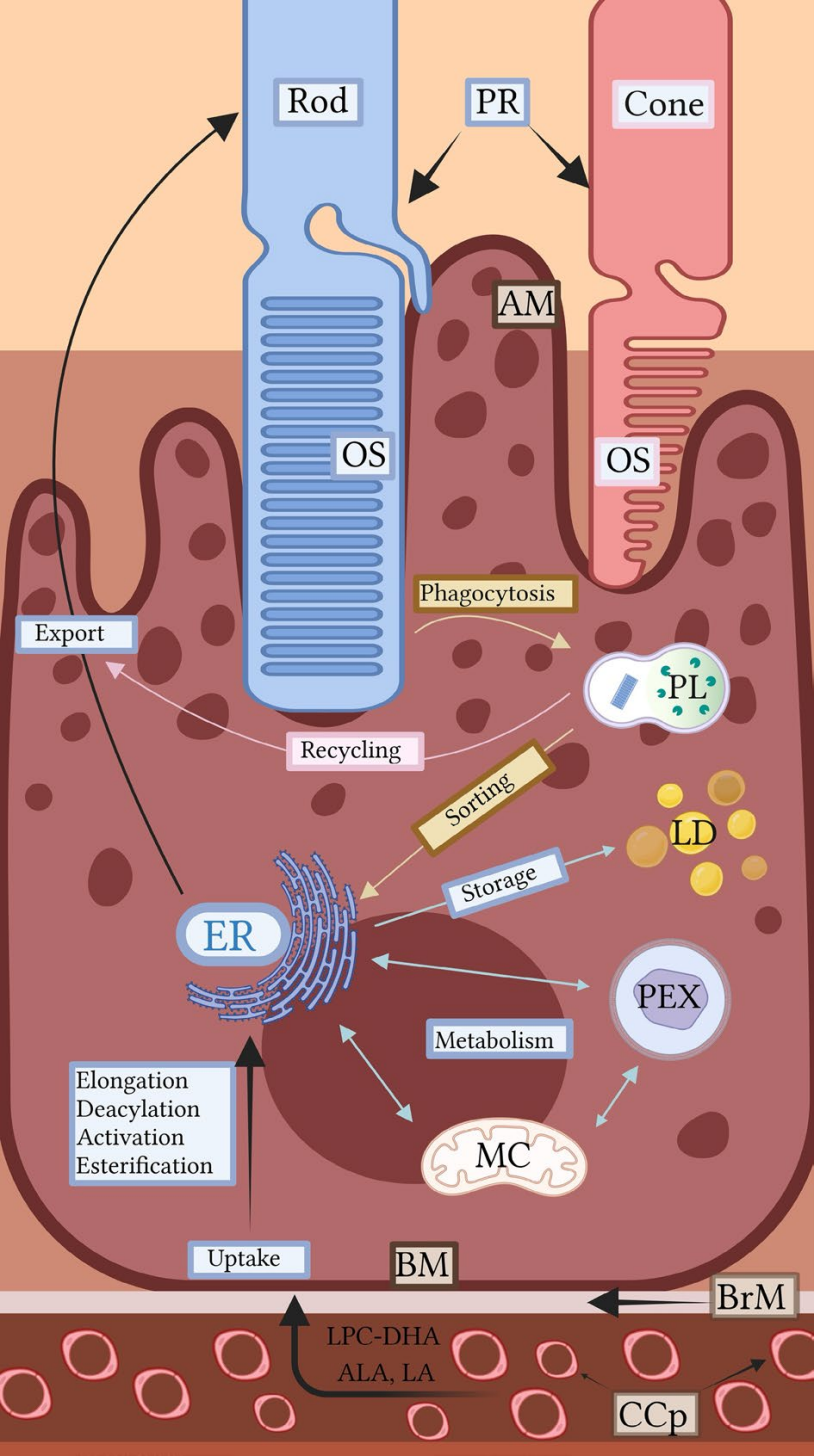
This schematic illustrates the coordinated uptake, metabolism and export of PUFAs in the RPE. The RPE transports PUFAs from the systemic circulation through choriocapillaris (CCp) transported across the Bruch’s membrane (BrM) and basal membrane (BM) and acquires PUFAs at the apical membrane (AM) through RPE phagocytosis of photoreceptor (PR) outer segments (OS). Following the phagolysosome (PL) activity, a pool of PUFAs is recycled back to photoreceptors and the remaining pool is sorted to the endoplasmic reticulum (ER). The ER activates PUFAs that enter the RPE from the systemic circulation and stores excess non-recycled PUFA into LDs. In the RPE, a coordinated interaction among the organelles: ER, LD, peroxisomes (PEX) and mitochondria (MC) regulates PUFA metabolism. The mechanism of PUFA export to the retina remains an open area of investigation. “Created using BioRender.com” (https://BioRender.com/sh1gdkc)

In this review, we examine the genes and proteins that determine PUFA influx and trafficking into the RPE from the systemic circulation and the retina; PUFA metabolism within the RPE; and PUFA efflux from the RPE to the retina, as summarized in Fig. 1. We highlight these molecular players in the context of organelles, including the endoplasmic reticulum, lipid droplets (LDs), peroxisomes, and mitochondria that work in a coordinated fashion to maintain PUFA homeostasis. Where possible, we draw on literature that specifically examines RPE PUFA metabolism, but when direct evidence is unavailable, we infer plausible molecular functions based on PUFA studies in other tissues, cells, or model systems. The effects of mutations on molecular players are discussed and disease consequences of dysregulated PUFA metabolism, known and potential, are considered. Through this review, we hope to promote a greater understanding of the molecular pathways in the RPE that regulate retinal PUFA levels, providing insight into ocular diseases that are linked to genetic defects in PUFA metabolism, and possibly aid in identifying targets for therapeutic intervention.

## PUFA influx into the RPE

The RPE has evolved an array of highly coordinated networks of metabolic and trafficking mechanisms to regulate PUFA influx. Broadly, PUFA influx into the RPE occurs through two major pathways: 1) uptake from the systemic circulation, through basal receptors (Fig. 1 [uptake]), and 2) uptake from phagocytosed POS tips (Fig. 1 [recycling]). Because RPE consists of polarized epithelial cells containing distinct apical and basolateral domains, the following section focuses on molecules involved in the uptake and transport of PUFAs on both the apical and basolateral surfaces. Intracellular chaperones, which shuttle PUFAs to organellar destinations within the RPE cytoplasm are also discussed.

### Basal uptake

The RPE acquires and processes ω-3 and ω-6 PUFAs, such as DHA, ALA, AA and LA, from the choroidal blood supply in either a free FA or esterified form *e.g.* lysophosphatidylcholine-docosahexaenoic acid (LPC-DHA). Dietary studies, in which animal models were subjected to PUFA-deficient diets, show retinal morphological alterations and reduced ERG responses, underscoring the importance of PUFAs for retinal integrity and function (Anderson et al. 2005; Harrison et al. 2002; Jeffrey and Neuringer 2009; Weisinger et al. 1998). In vitro analyses in human ARPE-19 cells show temperature-dependent PUFA uptake, indicating the involvement of an energy-dependent transport process of PUFAs (Tachikawa et al. 2018). Lipid transporters such as MFSD2A, FA transporter proteins (SLC27A1 through 6, also known as FATP1-6) and lipoprotein receptors (LDLR, SCARB1) have been studied and are considered to play roles in moving PUFAs across the basal RPE plasma membrane.

#### MFSD2A

MFSD2A is an integral membrane transporter that transports esterified PUFAs, such as LPC-DHA, with high affinity in a sodium-dependent manner (Wong et al. 2016). Studies have shown a ~ 40% reduction in DHA levels in the eyes of *Mfsd2a* knockout mice compared to controls, indicating that it is a major transporter of DHA in the eye (Lobanova et al. 2019; Wong et al. 2016). Available evidence suggests that sn-2 lysophospholipid forms of DHA and precursors are taken up by the eye in a highly efficient manner. Animal diet studies showed little or no change in retinal DHA when free DHA or triacylglycerol-DHA was administered. However, when LPC-DHA or LPC-EPA was used, the highest levels of retinal DHA were achieved (Sugasini et al. 2020). Although MFSD2A variants have not been directly associated with visual impairment, multiple families with biallelic variations in MFSD2A have been reported with severe congenital microcephaly (Table 1) (Guemez-Gamboa et al. 2015; Harel et al. 2018; Khuller et al. 2021; Scala et al. 2020), a condition that is sometimes associated with chorioretinopathy as seen in several genetic disorders (Martín-Rivada et al. 2021; Thomas-Wilson et al. 2023). However, the full extent of ocular changes in such patients remains to be explored. More detailed information is available from studies of *Mfsd2a* knockout mouse models, which exhibit a specific reduction in PR outer segment length, disordered POSs, and accumulation of immune cells in the subretinal space (Lobanova et al. 2019; Park et al. 2021; Wong et al. 2016).

**Table 1:** Lipid transporters, function and associated disease and models

GENE	OMIM #	Function	Diseases	Human Ocular phenotypes	Biochemical alterations human*/mouse^#^	Mouse models retinal/ocular phenotypes
Pufa transporters and Chaperones						
*MFSD2A*	614397	Transports long chain FAs in the esterified form like LPC-DHA in a sodium dependent manner (Nguyen et al. 2014)	Microcephaly, Intellectual disability (MIM:616486) (Khuller et al. 2021)	PR degeneration, drusen deposits (Scala et al. 2020)	^#^Around 40% decrease in retinal DHA supply (Wong et al. 2016)	*Mfsd2a*^*tm1Dls*^ (MGI:5583066) Homozygotes show incomplete postnatal lethality (Berger et al. 2012), abnormal outer retina segments, autofluorescence in POS (Lobanova et al. 2019; Wong et al. 2016)
*SLC27A1*	600691	SLC27A1 (FATP1), functions as an acyl-CoA synthetase that interacts with PUFAs (Hall et al. 2003). SLC27A1 interacts with RPE56 and lectin, regulates 11-cis retinol formation (Guignard et al. 2010). May play a role in cellular lipid accumulation (Huang et al. 2021)	SLC27A1 – metabolic disorders (Huang et al. 2021)	No human retinal phenotype reported	^#^Decreased ERG response to light, due to the PR dysfunction (Chekroud et al. 2012)	*Slc27a1*^*tm1a(EUCOMM)Hmgu*^ (MGI:5294178) Reduced ERG responses in homozygous KO mice, with aging disorganization of OS, Bruch’s membrane thickening, choroidal vascular abnormalities (Chekroud et al. 2012)
*SLC27A4*	604194	SLC27A4 (FATP4) functions as acyl-CoA synthetases with specificity for very long-chain FAs (VLCFA). Negative regulator of RPE65 (Li et al. 2013)	Ichthyosis prematurity syndrome (Klar et al. 2009). (MIM:608649)	No human retinal phenotype reported	^#^Increased accumulation of the cytotoxic all-trans retinaldehyde and hyper susceptibility to light-induced PR degeneration (Li et al. 2013) Loss of *Slc27a4* in the retina of *rd12* mice increases levels of 9-cis-retinal and reduces M-cone degeneration (Li and Jin 2024)	*Slc27a4*^*tm1.1Wsr*^ (MGI:2667329) PR degeneration (Li et al. 2013)
*CD36*	173510	A scavenger receptor for oxidized phospholipids derived from the common retinal FA DHA, activated under oxidative stress (Sun et al. 2006)	Coronary heart disease (MIM:610938), Platelet glycoprotein IV deficiency (MIM:608404)	No human retinal phenotype reported	^#^Oxidized lipid accumulation and heightened susceptibility to oxidized lipid driven inflammation and accumulation of subretinal deposits (Picard et al. 2010)	*Cd36*^*tm1Mfe*^ (MGI:1931790) Subretinal deposits, Bruch’s membrane thickening (Picard et al. 2010)
*ABCA1*	600046	ATP binding cassette transporters ABCA1 efflux lipids from cell to APOAI and HDL, helping to prevent lipid overload (Cavelier et al. 2006)	ABCA1 -Hypoalphalipoproteinemia (MIM:604091), Tangier disease (MIM:205400)	ABCA1-Corneal opacity, ectropion, the mottled retinal pigment in the macula and/or periphery (Pressly et al. 1987)	^#^Impaired ABCA1/ABCG1 leads to neutral lipid accumulation as LDs in RPE leading to reduced RPE and retina function (Storti et al. 2019)	*Abca1*^*tm1.1jp*^ (MGI:3577725), *Abcg1*^*tm1Tall*^ (MGI:5450880) Retinal degeneration and RPE structural abnormalities (Ban et al. 2018; Storti et al. 2019; Westerterp et al. 2012; Yvan-Charvet et al. 2007)
*MTTP*	157147	Microsomal TG transfer protein (MTP) essential for the biogenesis of APOB containing lipoproteins (Hussain et al. 2012)	Abetalipo-proteinemia (MIM:200100)	Progressive retinal dystrophy (Alshareef et al. 2015)	^#^Lipid accumulation in PLIN2 positive aggregates in RPE and cholesterol aggregates in retina (Grubaugh et al. 2024)	*Mttp*^*tm1sgy*^ (MGI:2152201), *Mttp*^*tm1Chan*^ (MGI:2386838) *RPEΔMttp* conditional mice show retinal degeneration, subretinal deposits, hyperreflective foci, increased LD accumulation in the RPE (Grubaugh et al. 2024)
*LDLR*	606945	Cell surface receptor that internalizes cholesterol and/or free FA rich lipoproteins from extracellular fluids, usually binds to ApoB-100 on LDL particles (Murakami et al. 1990)	Familial Hypercholesterolemia (MIM:143890)	Corneal arcus, retinal atherosclerosis and xanthomas (Bou Ghannam et al. 2024; Meng et al. 2013)	^#^Reduced ERG response, scotopic and photopic b-wave with age, cholesteryl ester accumulation at the basement of RPE (Bretillon et al. 2008). Accumulation of neutral lipids in BM (Schmidt-Erfurth et al. 2008)	*Ldlr*^*tm1Her*^ (MGI:1857212) Thickening of Bruch’s membrane (BM), BM lipoidal degeneration, RPE atrophy, disorganized OS discs (Schmidt-Erfurth et al. 2008)
*ADIPOR1*	607945	Transmembrane protein, adiponectin as ligand. Involved in induction of insulin resistance and glucose intolerance. Anti-inflammatory and pro cell survival factor (Yamauchi and Kadowaki 2013)	Syndromic retinitis pigmentosa, truncal obesity (single case) (Xu et al. 2016). Autosomal dominant retinitis pigmentosa (segregation in family) (Zhang et al. 2016) Association with severe AMD (Kaarniranta et al. 2012)	Extensive pigmentary retinopathy, tunnel vision, cataracts, advanced waxy optic nerve pallor (Xu et al. 2016)	^#^Marked reduction of ω-3 PUFAs and elevation of ω-6 PUFA in retina and RPE. Also increase in sphingolipids, ceramide species, and decrease in acylcarnitines in retina and in RPE increase in phosphatidylcholine, cholesterol ester and lyso-phosphatidylcholine (Lewandowski et al. 2024)	*Adipor1*^*tm1Dgen*^ (MGI:3604517) Early loss of outer segment and flecked retina (Osada et al. 2021; Sluch et al. 2018), microphthalmia (Gogna et al. 2025)
*MFRP*	606227	Integral membrane protein with frizzled family domain, two CUB domains, LDLR domain and cysteine rich domain. Expressed as dicistronic transcript with *C1qtnf5.* Located on the apical surface of RPE and considered a molecular hub (Kameya et al. 2002; Tworak et al. 2025)	Microphthalmia (MIM: 611040), Nanophthalmos (MIM: 609549), Retinitis pigmentosa (Vanden Heuvel et al. 2023)	Short axial length, progressive retinal dystrophy, foveoschisis, optic disc drusen (Ayala-Ramirez et al. 2006)	^#^Decreased AA/DHA ratio in RPE and increased AA/DHA ratio in retina (Tworak et al. 2025)	*Mfrp*^*rd6*^ (MGI:1888705), *Mfrp*^*rdx*^ (MGI:5312653) White retinal spots and photoreceptor degeneration (Hawes et al. 2000; Kameya et al. 2002) and microphthalmia (Gogna et al. 2025). Progressive retinal degeneration, RPE atrophy, fundus flecks (Fogerty and Besharse 2011)
Pufa desaturases and elongases						
*FADS1*	606148	Encodes Δ5-Desaturase Introduces double bonds between defined carbons of the fatty acyl chain. Converts 20 carbon PUFA intermediates into Arachidonic acid (20:4n-6) and Eicosapentaenoic acid (20:5n-3)	Haplotypes are linked to variations in LC-PUFA synthesis (Ameur et al. 2012). Association with lifestyle related diseases (Sergeant et al. 2012)	No human retinal phenotype reported	^#^AA depletion, leading to downregulation of eicosanoid synthesis, reduced signaling and secondary molecules like phosphoinositide species (Sergeant et al. 2012)	*Fads1*^*tm1.1(KOMP)Vlcg*^ (MGI:5695931), *Fads1*^*Gt(IST11525H2)Tigm*^ (MGI:5147023) Premature lethality. Abnormal lipid levels. No eye phenotypes reported (Fan et al. 2012)
*FADS2*	606149	Encodes Δ6-Desaturase. Rate limiting step in introducing a double bond to 18-carbon precursors like linoleic acid (LA) and alpha linoleic acid (ALA)	Selected in human lineage (Ameur et al. 2012). Association with lifestyle related diseases (Khodarahmi et al. 2022; Roke and Mutch 2014; Shetty and Kumari 2021)	Corneal ulceration and photophobia, associated with FAs deficiency (Williard et al. 2001)	^#^Reduced levels of DHA biosynthesis, leading to LC-PUFA deficiency (Stoffel et al. 2008)	*Fads2*^*tm1Mtna*^ (MGI:4365366) Significant reduction in ERG scotopic amplitudes with DHA deficient diet (Chauhan et al. 2023)
*ELOVL2*	611814	Elongase for synthesis of VLCFAs, PUFA synthesis, conversion of C20-C22 PUFAs to C24-C26 PUFAs (Leonard et al. 2002). Epigenetic biomarker for systemic biological aging (Garagnani et al. 2012)	Genetic variations associated with onset of intermediate AMD (Gao et al. 2025)	Early onset of intermediate AMD (Gao et al. 2025)	*Reduction of LC- and VLC-PUFAs and increase ratio of n-6/n-3 PUFAs in retina and RPE/choroid of AMD donor eyes (Gordon et al. 2023; Liu et al. 2010) ^#^ER stress and mitochondrial dysfunction. Progressive decline in DHA (22:6) and VLC-PUFA abundance in aged mice (Gao et al. 2025)	*Elovl2*^*em1Haml*^ (MGI:6782853) Fundus spots by 6 months, loss of scotopic function (Chen et al. 2020; Gao et al. 2025)
*ELOVL4*	605512	Elongates LCFAs like C26 PUFAs to VLC-FAs like C28-C38 PUFAs (Agbaga et al. 2008)	Ichthyosis, spatial quadriplegia (MIM:614457) Spinocerebellar ataxia 34 (MIM:133190), Stargardt type3 (MIM:600110) (Zhang et al. 2001)	Stargardt type 3 associated with retinal degeneration distinguished by early onset central vision loss, progressive macular dystrophy, macular flecks and progressive loss of foveal rod response (Zhang et al. 2001)	^#^Accumulation of lipofuscin in RPE, Significant lower levels of FAs (Vasireddy et al. 2006). Increased amount of A2E precursors (McMahon et al. 2007)	*Elovl4*^*tm1Rayy*^ (MGI: 3692750), *Elovl4*^*tm1Wked*^ (MGI:3711216), *Elovl4*^*tm1Kzh*^ (MGI:3722127) Homozygous null mice show perinatal lethality. Heterozygotes have progressive retinal degeneration, subretinal deposits (Vasireddy et al. 2006). Reduced rod ERG amplitudes (McMahon et al. 2007)
Esterifying & acylation agents						
*ACSL6*	604443	Catalyzes the conversion of long chain free FAs to acyl CoA before their incorporation into membrane phospholipids or β-oxidation. High affinity interaction with DHA (Fernandez et al. 2018)	Association with Premature ovarian failure (Kang et al. 2009)	No human retinal phenotype reported	^#^Loss of di-DHA PC needed for retina outer segments formation (Wang et al. 2024)	*Acsl6*^*tm1.1Jeme*^ (MGI: 6268795) Fundus spots by 6 months of age, declining scotopic ERG response and progressive loss of rod PRs (Fernandez et al. 2021; Wang et al. 2024)
*AGPAT3*	614794	Lysophosphatidic acid acyltransferase gamma (LPAAT3). Member of acyltransferase family that catalyzes the acylation of lysophosphatidic acid to form phosphatidic acid	Intellectual disability and retinitis pigmentosa syndrome (Malik et al. 2023)	Loss of outer segments, cataract (Malik et al. 2023)	^#^Reduction of PL-DHA in retina, increase in PL-AA rather than PL-DHA in PR (Shindou et al. 2017)	*Agpat3*^*tm1a(EUCOMM)Wtsi*^ (MGI:4432492) Impaired visual function, disordered PR disk morphology, loss of outer segments (Shindou et al. 2017)
*LPCAT1*	610472	Phospholipid biosynthesis/remodeling enzyme that preferentially transforms palmitoyl-CoA to LPC and is part of lipid remodeling pathway (Nagata et al. 2022)	No mendelian disease known	No human retinal phenotype reported	Reduced dipalmitoyl-PC in retina, loss of outer nuclear layer, increased mitochondrial reactive oxygen species (Friedman et al. 2010)	*Lpcat1*^*rd11*^ (MGI:2388243) Rapid photoreceptor degeneration (Friedman et al. 2010; Nagata et al. 2022)
Lipase activity						
*PNPLA2* *(PEDF-R)*	609059	Expressed in photoreceptor cells and RPE. PEDF binding partner secreted by RPE stimulates its PLA2 activity (Notari et al. 2006). Liberates FAs from phospholipids, specifically those with DHA in sn-2 position (Bernardo-Colon et al. 2023)	Neural lipid storage disease with myopathy (MIM: 610717) (Tavian et al. 2021)	No human retinal phenotype reported	^#^Decreased PLA2 activity, rhodopsin and opsin in retina, increased LPC-DHA and LPE-DHA in retina, lipid accumulation (Bernardo-Colon et al. 2023; Bullock et al. 2021)	*Pnpla2*^*tm1.1Hsul*^ (MGI:5296237) Conditional *RPEΔPnpla2*, impaired phagocytosis (Bullock et al. 2021), *Pnpla2*^*em1Dong*^ (MGI:8282380) Abnormal PR morphology, retinal degeneration and abnormal phospholipid composition (Bernardo-Colon et al. 2023), *Pnpla2*^*tm1Rze*^ (MGI:3629035) accumulation of LDs in RPE, reduced ERG responses (Hara et al. 2023)
*PNPLA6* *(NTE)*	603197	*PNPLA6* (neuropathy targeting esterase) catalyzes the PLC reaction, removing 2 fatty acyl chains from the sn-1 and -2 positions of PC and LPC to generate GPC	*PNPLA6* related disorders (MIM: 275400), (MIM:245800), (MIM:215470), (MIM:612020), Boucher-Neuhauser syndrome, Gordon–Holmes syndromes (O’Neil et al. 2019)	Chorioretinal atrophy, syndromic retinal dystrophy, retinal degeneration (Wu et al. 2021)	^#^Elevated PC, LPC levels of saturated and unsaturated including PUFA in RPE of knockout mice. RPE oxidative stress, photoreceptor dysfunction leading to retinitis pigmentosa (Ono et al. 2025)	*Pnpla6*^*tm1Murm*^ (MGI: 8282391) *Pnpla6*^fl/fl^*Best1-Cre* (ΔRPE) Reduced ERG amplitudes, retinal thinning *Pnpla6*^fl/fl^*CAG-CreER* (ΔPR) Thinning of outer nuclear layer and RPE, reduced RPE microvilli and mitochondria, retinal degeneration (Ono et al. 2025)
*PLA2G5*	601192	Hydrolyzes the ester bond of the fatty acyl group attached at sn-2 position of phospholipids (Sergouniotis et al. 2011)	Familial benign flecked retina (MIM:228980)	Diffuse yellow white retinal lesions (Sergouniotis et al. 2011)	*Presence of hyper autofluorescent material rich in lipofuscin like material in the RPE (Sergouniotis et al. 2011)	*Pla2g5*^*em1(IMPC)Ccpcz*^ (MGI:6388359) No eye phenotype reported
*LIPA*	613497	Lysosomal acid lipase (LAL) encoded by *LIPA* gene, functions in the lysosomes to catalyze the hydrolysis of cholesterol esters and TGs. In the RPE lysosomes are upregulated in the presence of ω-3 PUFA intake facilitating hydrolysis of PUFA containing cholesterol and TGs derived from POS(Elner 2002)	Wolman disease (MIM:620151) and cholesterol ester storage disease (MIM:278000) (Saito et al. 2012)	No human retinal phenotype reported	*Increased RPE LDs, decreased RPE function, reduced rod photoreceptor function in patients (Lee et al. 2025)	*Lipa*^*tm1a(EUCOMM)Hmgu*^ (MGI:5428633), *Lipa*^*tm1Hodu*^ (MGI:2451080) In both global KO and conditional (ΔRPE) on high fat diet, accumulation of LDs in RPE and reduced ERG function (Lee et al. 2025)

#### SLC27A (FATP)

The solute carrier 27A gene family in humans encodes FA transporter proteins (SLC271A1–6, also known as FATP1–6). These dual-function integral membrane proteins mediate the transport of PUFAs across the plasma membrane and the linkage of PUFAs to acetyl-CoA by a long-chain fatty acyl-CoA synthetase activity (Ochiai et al. 2017; Tachikawa et al. 2018). This association constitutes a “vectorial acylation” resulting in efficient capture and retention of PUFAs within the cell. SLC27A4 is predominantly found in the RPE, exhibits very long chain acyl-CoA synthetase activity, and contributes to PR homeostasis, by regulating lipid metabolism (Li et al. 2013; Tachikawa et al. 2018). While no ocular phenotype has been reported in patients carrying mutations in members of the *SLC27A* gene family, RPE-specific ablation of *Slc27a4* in mice leads to impaired lipid handling and increased susceptibility to light-induced PR degeneration (Li et al. 2013). *Slc27a1* is also abundantly expressed in the RPE, where it facilitates FA uptake and plays a role in retinoid metabolism (Cubizolle et al. 2017) (Table 1). RPE-specific overexpression of human *SLC27A1* in mice increases FA uptake and lipid metabolic activity, leading to increased retinoid ester accumulation and increased susceptibility to light induced PR degeneration (Cubizolle et al. 2017). In summary, while in vitro and in vivo animal studies provide evidence that SLC27A family members are likely to play a central role in FA and PUFA uptake by the RPE, we do not have a clear understanding of the retinal disease phenotypes that might occur as a result of PUFA imbalance due to *SLC27A1–6* variation in humans.

#### Lipoprotein receptors

RPE cells express a network of lipoprotein receptors to acquire PUFAs in lipoproteins from the systemic circulation (Edmond 2001; Elner 2002; Tachikawa et al. 2018; Wang and Anderson 1993). These receptors include the low density lipoprotein receptor (LDLR) and other members of the LDLR family, such as very low density lipoprotein receptors (VLDLR) (Vasandani et al. 2002), scavenger receptor class B type 1 (SCARB1) (Spady et al. 1999) and CD36 (Alexander Aguilera et al. 2006; Okamoto et al. 1998). LDLRs internalize lipoproteins through clathrin-mediated endocytosis, delivering PUFA-containing cholesteryl esters and triglycerides (TG) into the RPE through lipoprotein particles (Gordiyenko et al. 2004). LDLR and VLDLR bind APOB and APOE present on LDL and VLDL particles (Blacklow 2007). Internalized LDL particles are delivered to lysosomes where cholesteryl esters and TG are hydrolyzed by RPE lysosomal acid lipase, releasing cholesterol and free FAs such as PUFAs (Elner 2002). Notably, diets rich in ω-3 PUFA enhance the delivery of DHA via LDL particles and promote lysosomal lipid processing in the RPE, thereby potentially reducing lipofuscin accumulation and oxidative stress (Elner 2002). Based on the above activities, a deficiency of LDLR or VLDLR in the RPE might be expected to alter PUFA levels. While we have not identified published lipidomic evidence demonstrating altered PUFA levels in RPE-specific *Ldlr* and *Vldlr* knockout mice, analysis of the retina in *Vldlr* knockout mice showed a reduction in medium- and long-chain FAs (Bretillon et al. 2008; Joyal et al. 2016). Similarly, although direct evidence of altered RPE PUFA levels in *Apoe* or *Apob* mutant mice is limited, genetic linkage and association studies in humans indicate that apolipoprotein-mediated lipid pathways influence retinal lipid homeostasis and may indirectly affect PUFA levels in the RPE (Curcio et al. 2010; Klaver et al. 1998; Souied et al. 1998; Vandal et al. 2014).

The SCARB1 receptor in RPE cells facilitates the selective uptake of high-density lipoprotein (HDL) derived lipids including cholesteryl esters and macular carotenoids (Li et al. 2023). *Scarb1* knockout mice, fed a normal chow diet, do not show an abnormal retinal phenotype. However, administration of a high fat and cholesterol-containing diet leads to PR layer disorganization in these mice, as well as accumulation of subretinal LDs and significant thickening of Bruch’s membrane (Duncan et al. 2002; Provost et al. 2009), further supporting the role of SCARB1 in retinal cholesteryl ester handling and lipid homeostasis (Shen et al. 2018). This transporter may also provide a potential route for PUFA uptake via transport of PUFA-cholesteryl esters. Although there is evidence for participation of lipoprotein receptors in PUFA uptake in both human and mice, their precise role needs to be studied.

Altogether, these basal membrane uptake pathways establish the RPE as a selective gateway for systemic PUFA uptake into the outer retina.

### Apical transport

The apical surface of the RPE represents a uniquely specialized interface dedicated to sustaining PR lipid homeostasis. While some proteins on the apical surface of the RPE act as regulators for the transport of PUFAs to PRs (Rice et al. 2015), others are engaged in the daily phagocytic import of POS tips into the RPE (Kwon and Freeman 2020). Importantly, these apical phagocytic molecules along with the transporters act as a hub for recycling PUFAs obtained from POS phagocytosis.

### PUFA regulators

#### ADIPOR1

The adiponectin receptor 1 (ADIPOR1), a regulator of PUFA homeostasis, is expressed on the apical surface of the RPE (Sluch et al. 2018). Mutations in *ADIPOR1* in humans cause syndromic and non-syndromic retinitis pigmentosa (Xu et al. 2016; Zhang et al. 2016), a phenotype similar to that observed in *Adipor1* null mice (Rice et al. 2015; Sluch et al. 2018). Lipidomic analysis of an *Adipor1* knockout mouse showed a marked reduction in ω-3 PUFAs and accumulation of ω-6 PUFAs in the RPE and retina, reduced VLC-PUFA-containing phosphatidylcholine species in PRs, and ceramide elevation (Lewandowski et al. 2022a; Rice et al. 2015) along with progressive PR degeneration (Gogna et al. 2025, 2022; Lewandowski et al. 2022a; Rice et al. 2015). While some of these phenotypes may also be attributable to the loss of *Adipor1* expression in retinal cells, an increase in DHA levels in TG is also seen in the RPE of *Adipor1* knockout mice. This increase is consistent with altered handling and retention of DHA within the RPE rather than its efficient transport to PRs, highlighting the role of ADIPOR1 in regulating ω-3 PUFA transport from the RPE. Administration of ceramidase inhibitors improved retinal function and elevated ω-3 PUFAs in the retina and RPE of *Adipor1* KO mice, suggesting a potential therapeutic strategy for DHA deficiency (Lewandowski et al. 2024). Collectively, these findings identify ADIPOR1 as a critical RPE apical regulator that maintains lipid levels in the outer retina and its role in retinal homeostasis and PUFA regulation warrants further investigation.

### MFRP

Membrane frizzled-related protein (MFRP) is expressed prominently at the apical surface of RPE cells (Kameya et al. 2002; Won et al. 2008). A spectrum of mutations and ocular phenotypes such as posterior microphthalmos, high hyperopia, foveoschisis, and retinitis pigmentosa have been observed in humans (Mukhopadhyay et al. 2010; Vanden Heuvel et al. 2023). Transcriptomic and lipidomic analysis of *Mfrp*^*rd6*^ mutant mice has shown that MFRP may play a role in lipid homeostasis, which includes the enrichment and distribution of PUFAs (Kautzmann et al. 2020; Tworak et al. 2025). Lipidomic profiling of *Mfrp*^*rd6*^ mice identified a reduction in lipid biosynthesis and imbalance in overall lipid homeostasis in the RPE. Specifically, an accumulation of DHA with a significant reduction of the n-6:n-3 PUFA ratio is seen in the RPE. In contrast, a > threefold increase in the n-6:n-3 PUFA ratio is observed in the retina of *Mfrp*^*rd6*^ mice relative to controls (Tworak et al. 2025). Related disturbances in PUFA distribution and lipid homeostasis have also been reported in the RPE and retina of *Adipor1* knockout mice (Sluch et al. 2018). The question arises: is the altered PUFA homeostasis directly related to loss of MFRP in *rd6* mice or is it due to the dysregulation of PUFA regulators such as MFSD2A, ADIPOR1, ACSL6, and ELOVL family members, which are also affected in *Mfrp*^*rd6*^ mice (Tworak et al. 2025).

### RPE apical phagocytosis

To achieve the daily renewal and recycling of POSs, RPE apical processes recognize and engulf shed POS tips via a coordinated phagocytic process. RPE cell surface molecules αvβ5 integrin (Nandrot et al. 2004) and MERTK (Feng et al. 2002) mediate uptake of the POS tips into phagosomes for lysosomal digestion. Engulfing requires actin remodeling driven by RAC1 with regulatory contributions from proteins such as annexin A2 and kinase signaling molecules including AKT, which enable phagocytic cup formation and POS internalization (Mao and Finnemann 2012). Loss of phagocytic function leads to retinal degeneration, highlighting the importance of the processes and molecules in POS internalization (Chaitin and Hall 1983; D’Cruz et al. 2000; Maddox et al. 2011). Following internalization, POS-laden phagosomes undergo directed trafficking supported by myosin VIIa (Gibbs et al. 2003), followed by transfer to microtubules via kinesin and dynein-mediated apical-to-basal movement. Basal phagosomes mature and fuse with lysosomes, where the POS cargo is degraded (Lakkaraju et al. 2020; Wavre-Shapton et al. 2014). The presence of high DHA content renders POS highly sensitive to oxidation. Oxidized lipids are captured by CD36, where CD36 recognizes these oxidized lipids through its extracellular hydrophobic binding pocket that has a high affinity for oxidized phospholipid epitopes (Sun et al. 2006). Accumulation of oxidized lipids can contribute to RPE cell dysfunction and death and may play a role in AMD, as oxidized lipids are found to accumulate in the aging macula (Gnanaguru et al. 2016; Suzuki et al. 2007).

RPE lipases release FA including PUFAs from phospholipids present in phagocytosed POS. Phospholipase A2s (PLA2s) comprise a large lipase family in humans with 29 members that include secretory, cytosolic, and calcium-dependent forms (Burke and Dennis 2009; Kolko et al. 2007; Murakami et al. 2011). PLA2s cleave ester bonds at the sn-2 position of membrane phospholipids, including PUFA-rich phospholipids obtained through the phagocytosis of PRs. The activation of PLA2s is influenced by the metabolic state of the RPE. For example, ischemia, light exposure, oxidative stress, apoptosis, inflammation and aging may affect PLA2 activity (SanGiovanni and Chew 2005). Human patients with mutations in the *PLA2* family member, *PLA2G5* show defects in RPE phagocytosis that lead to the formation of auto-fluorescent yellow-white flecks in the retina (Sergouniotis et al. 2011). Another highly expressed RPE lipase, PNPLA2, also exhibits PLA2 activity with a high preference for DHA in the sn-2 position of phagocytosed POS lipids (Bullock et al. 2021; Subramanian and Becerra 2019). *PNPLA2* silencing using siRNA treatment in ARPE-19 cells abolished digestion of POS rhodopsin and PUFA-rich lipids (Bullock et al. 2021). Moreover, RPE-specific *Pnpla2* knockout mice accumulated LDs in the RPE, consistent with an in vivo role of PNPLA2 in lipid processing. Thus, PNPLA2 lipase activity in the RPE may be central to releasing PUFAs from ingested POS (Bullock et al. 2021).

Under normal conditions, following the lipolysis and release of DHA from phagocytosed POS, the RPE either recycles DHA for new membrane synthesis (Rice et al. 2015) or breaks it down via ꞵ-oxidation for energy generation (Adijanto et al. 2014). Under oxidative stress conditions, the DHA that is released is converted into neuroprotectin D1 (NPD1), a docosanoid mediator that helps in cell survival (Mukherjee et al. 2007). Although the precise fate of DHA released from phagocytosis is unknown, a possible pathway is transport to the ER and rapid esterification into TG, which subsequently coalesce into LDs (Wilfling et al. 2013).

### Intracellular PUFA trafficking/chaperones

#### Fatty acid binding proteins (FABPs)

FA binding proteins (FABP) are cup-shaped soluble proteins that have high binding affinity for FAs (saturated and unsaturated) and play a major role in chaperoning FAs within the cytoplasm to various cellular compartments, such as the mitochondria, peroxisomes, nucleus, endoplasmic reticulum, and LDs (Furuhashi and Hotamisligil 2008; Umetsu et al. 2022). FABP5 has been studied extensively in the RPE (Ohguro et al. 2025), where the protein is strongly linked to DHA and AA trafficking. Knockdown of *FABP5* in ARPE-19 cells results in a decrease in FA uptake, which might disrupt intracellular transport of FAs leading to an accumulation of FA-containing LDs (Wu et al. 2010). Studies in the brain and spinal cord have further demonstrated roles for FABPs in PUFA uptake, transport and metabolism. Elevated expression of FABP5 facilitated ω-3 PUFA metabolism and attenuated secondary damage, promoting functional recovery of post spinal cord injury and neurotrauma conditions (Figueroa et al. 2015). *FABP4/Fabp4* is also expressed in human and rodent RPE (Ohguro et al. 2024). Alteration and mutations affecting PUFA regulation could potentially reshape FABP abundance or function as seen in, in vitro and in vivo models. Knockdown of peroxisome proliferator-activated receptor γ coactivator 1α (*PGC1A)* in ARPE-19 cells, downregulated *FABP4* expression, impairing the uptake of exogenous FAs (Zhou et al. 2024). Similarly, the RPE of *Adipor1*^*−/−*^ mice showed a significant downregulation of *Fabp1* levels and FABP3 abundance, respectively, highlighting their role in PUFA metabolism and transport (Lewandowski et al. 2024). Alongside the RPE, FABP4 is abundantly detected in retina (Umetsu et al. 2022). FABP4 binds a broad range of long chain FAs with high affinity for unsaturated FAs like LA, AA and DHA, shuttling FAs to LDs, mitochondria and peroxisomes for oxidation, functions that it likely also carries out in the RPE (Furuhashi 2019).

## PUFA metabolism within the RPE

Imported PUFAs and precursors are metabolized in the RPE by several processes to ensure sufficient PUFA pools for maintaining retinal homeostasis: dietary precursors must be modified; phagocytosed PUFAs must be recycled or stored, and excess PUFAs may be used as a substrate for fuel. These processes are carried out by RPE organelles.

### Endoplasmic reticulum (ER)

Dietary essential FAs, LA and ALA, and their long‑chain derivatives AA and DHA (Sinclair et al. 2022), are sequentially modified to form LC-PUFAs. PR cells are unable to perform these functions, which instead are carried out in the ER of the RPE. Following uptake of the PUFAs from the systemic circulation, PUFAs like LA and ALA require elongation, desaturation, and acylation to yield active forms of LC- and VLC-PUFAs. The RPE ER is equipped with enzymes for FA activation. In brief, these steps involve the elongation, desaturation and acylation of the ω-3 or ω-6 PUFAs by enzymes such as FADSs (FA Desaturases), ELOVLs (Elongases or Elongation of Very Long chain FAs) and ACSLs (Long chain acyl-CoA synthetases).

Apart from preprocessing and activating PUFAs for transport from the RPE, the ER is also involved in the sorting and metabolic processing of PUFAs liberated from POSs in phagolysosomes. Based on studies of cultured rat RPE cells, POS-derived DHA appears to be transiently sequestered into TG (Rodriguez de Turco et al. 1999). Metabolic labeling experiments in frogs showed that a portion of phagocytosed POS DHA was distributed to RPE LDs, which were heavily labeled (Gordon and Bazan 1993). These results suggest a possible scenario in RPE cells, in which phagocytosed POS lipids, including PUFA-containing lipids, are delivered to the ER, where TGs are synthesized and then delivered to LDs. Although details of this process in the mammalian RPE are lacking, studies of the model organism *Caenorhabditis elegans* have revealed that an SLC27A family member and diacylglycerol acyltransferase DGAT2 coordinate at the ER-LD interface to esterify FAs to TGs, which are delivered to budding LDs (Xu et al. 2012). Related proteins in the human and mouse RPE may act similarly to incorporate phagocytosed PUFA in the form of TGs and LDs. ER-generated TG LDs are considered as storage facilities for FAs, including PUFAs (Danielli et al. 2023; Rodriguez de Turco et al. 1999).

#### Desaturases

FADS1 (Δ5-desaturase) and FADS2 (Δ6-desaturase) catalyze key desaturation steps in the biosynthesis of LC-PUFAs (Glaser et al. 2010). In a key and often rate-limiting step of converting LA and ALA into LC-PUFA, FADS2 introduces a double bond into the 18-carbon precursors. Further downstream, the related desaturase FADS1 converts 20-carbon PUFA intermediates into AA and EPA. Through its control of AA and EPA synthesis, FADS1 regulates downstream eicosanoid pathways (Gromovsky et al. 2018). Despite these important functions of FADSs, no RPE- or retina-specific disease phenotypes have been reported with loss-of-function mutations in FADS1/2. However, early-stage AMD is associated with an upregulation of FADS1/2, consistent with a possible compensatory or protective role of the SREBF1-FADS1/2 axis in early stage AMD (Ashikawa et al. 2017). Furthermore, pharmacological inhibition of FADS1 ameliorates RPE ferroptosis and dysfunction, suggesting a potential role of FA desaturases in RPE pathology (Zhang et al. 2025). Thus, while monogenic disruption of these genes has not been reported in humans, multiple lines of evidence suggest that variations in PUFA desaturation capacity influence RPE vulnerability and can be considered a therapeutic target for the treatment of AMD phenotypes.

#### Elongases

Along with desaturases like FADS, the elongation of LC- and VLC-PUFA in the RPE is controlled by ELOVL enzymes. ELOVLs are expressed to some extent in both RPE and PR cells. ELOVL1, 3, 6 and 7 are primarily involved in the elongation of saturated and monounsaturated FAs and have a limited role in PUFA metabolism (Deak et al. 2019). ELOVL2 and ELOVL5 show moderate to high expression in the RPE and contribute to the elongation of C18-C20 and C22-C26 PUFAs, respectively (Gregory et al. 2013). ELOVL2 is also considered a chronological age marker in the retina, based on age-related down regulation in ELOVL2 expression (Chen et al. 2020) and a correlation between ELOVL2 variants and the onset of intermediate AMD (Gao et al. 2025). No retinal degenerative disorders have been reported associated with mutations in ELOVL5. However, in clinical cases involving human ELOVL5 mutations spinocerebellar ataxias and nystagmus has been reported (Di Gregorio et al. 2014). Mouse global knock-out studies of Elovl5 showed decreases in EPA, docosapentaenoic acid (DPA) and AA levels (Moon et al. 2009), but no retinal phenotype or changes were reported. Mutations in ELOVL4 are associated with Stargardt-like macular dystrophy and autosomal dominant macular dystrophy (Zhang et al. 2001). ELOVL4 is not highly expressed in the RPE, and de novo synthesis of very‑long‑chain PUFAs in the RPE is thought to be limited compared with photoreceptors (Lagali et al. 2003). On the other hand, ELOVL4 is one of the most abundantly expressed elongases in rod and cone PRs where it elongates LC-PUFA such as C26 PUFAs to C28-C38 VLC-PUFAs in PR inner segments (Agbaga et al. 2008). These VLC‑PUFAs are recovered by the RPE through POS shedding and metabolism and subsequently contribute to photoreceptor lipid homeostasis.

#### Long chain acyl-CoA synthetases (ACSLs)

Upon RPE entry, LC-PUFAs are transferred to coenzyme A, forming an acyl-CoA thioester linkage that is activated for further metabolic reactions (Deng et al. 2025; Kuroha et al. 2023). Multiple long-chain fatty acyl-CoA synthetases are present at moderate to high levels in the RPE including ACSL1, ACSL3, ACSL4 and ACSL6 (Lewandowski et al. 2024). ACSL4 preferentially activates DHA and AA (Ding et al. 2023). ACSL4 also plays a key role in sensitizing RPE cells to ferroptosis by catalyzing the activation of LC‑PUFAs into PUFA‑CoA species, which serve as substrates for lipid peroxidation (Neiteler et al. 2023). ACSL6 activates PUFAs, especially DHA, and converts it into DHA-CoA which acts as a substrate during DHA-containing lipid biosynthesis (Kuroha et al. 2023). *Acsl6* retina-specific knockout studies show DHA retention behind the blood retinal barrier and altered PUFA composition which may be a pathological driver of the reported PR degeneration (Wang et al. 2024). Although less studied than ACSL4 and ACLS6 in the RPE, both ACSL1 and 3 are also found in the RPE. Under lipid metabolic stress conditions, *ACSL1* and *ACSL3* expression increases, indicating a compensatory or adaptive response to altered lipid metabolism (Lewandowski et al. 2024). Alongside the acyl-CoA synthetases, FA transporter proteins *SLC27A1* and *SLC27A4* in the RPE play an important role in PUFA acylation (Cubizolle et al. 2017; Li et al. 2020). Together, these acyl-CoA synthetases in the RPE work within an enzymatic network that controls PUFA-CoA availability for further metabolic processes, including lipid synthesis, elongation, and TG formation.

#### ER stress and PUFA metabolism

Chronic ER stress in the RPE can perturb PUFA metabolism at multiple levels and lead to deleterious effects. For instance, sterol regulatory element binding factor 1 (SREBF1) is a sensor that activates transcription of target genes, including *FADS1/2*, in response to cellular stress. In the RPE during early AMD, an initial protective response may activate the SREBF1-FADS2 axis to increase DHA synthesis (Ashikawa et al. 2017). However, prolonged activation of this axis may contribute to maladaptive changes. Studies have shown elevation of FADS1 in both an in vitro RPE stress model and an in vivo AMD mouse model (Zhang et al. 2025), suggesting that chronic ER stress in AMD can cause cells to produce more highly unsaturated FAs, promoting cellular sensitivity to ferroptosis (Lee et al. 2020a). Conversely, an inability to properly metabolize PUFAs can induce ER stress. For example, Bietti’s crystalline dystrophy (BCD, OMIM # 210370) is an inherited retinal degeneration caused by mutations in *CYP4V2,* a cytochrome P450 enzyme expressed in the RPE that oxidizes FAs. RPE cells with *CYP4V2* knockdown exhibit an unfolded protein response (UPR) activation due to upregulation of ER stress-related proteins in response to PUFA overload (Hsiao et al. 2025). This study demonstrated that in the presence of ER stress, surplus PUFAs are insufficiently metabolized, thus disrupting cellular homeostasis. In diseases like AMD, prolonged ER stress may lead to overproduction of pro-oxidative PUFAs and an insufficient ability to handle the oxidative load may promote cell death and inflammation. By targeting both the UPR and aberrant lipid metabolism, it may be possible to ameliorate ER stress and PUFA dysregulation, preserving RPE function. Future studies are needed to refine understanding of the relationship between PUFA metabolism and ER stress in the RPE.

### Lipid droplets

LDs were initially discovered in adipocytes as organelles that accumulate lipids and FAs (Fujimoto et al. 2008), and later in other cell types, such as brain microglia (Marschallinger et al. 2020). The core of LDs contains neutral lipids, predominantly TG and sterol esters (SE), surrounded by a phospholipid monolayer (Tauchi-Sato et al. 2002). LDs are present in the RPE, where they function as dynamic storage sites for neutral lipids and contribute to cellular lipid homeostasis (Orban et al. 2011). The RPE is capable of utilizing FAs derived from POS for β-oxidation and metabolic support, suggesting that LDs may serve as intracellular lipid reservoirs (Kocherlakota et al. 2023).

Following the engulfment of POSs containing PUFAs, they undergo degradation in lysosomes (Kwon and Freeman 2020). While studies in the retina are limited, research on non-ocular tissues, such as liver and intestine, have shown that FABP acts as an intracellular chaperone transporting FAs to the ER (Lagakos et al. 2011; Ohguro et al. 2024; Wu et al. 2010), where the pathways for the biosynthesis of TG and cholesteryl esters reside (Buhman et al. 2001; Wilfling et al. 2014). Enzymatic activities of GPAT3/4, AGPAT2, LPIN1 (Lipin-1) and DGAT1/2 are involved in the biosynthesis of TG, while ACAT is necessary for cholesteryl ester production (Wilfling et al. 2014; Xu et al. 2012). Once neutral lipids accumulate between the ER membrane leaflets and reach a critical concentration (2.8–10 mol%), seipin (BSCL2) and its binding partner, promethin (LDAF1) nucleate LDs at discrete ER subdomains, trapping TG molecules and promoting their phase separation into lens-like structures (Joshi and Cohen 2019; Klug et al. 2024). The nascent LDs then bud toward the cytoplasm through seipin’s cage-like structure, which maintains ER-LD contact sites to facilitate continued lipid transfer. The mature droplets are coated with perilipin proteins that protect droplets against lipolysis and regulate LD metabolism (Arlt et al. 2022). From this storage pool, FAs can be selectively mobilized by lipases and directed towards metabolic utilization.

LDs also associate with other organelles to form a dynamic organellar network to coordinate cellular lipid metabolism, energy production, and metabolic homeostasis. The LD coat protein, PLIN5, can bind to mitochondria and serve as a tethering bridge between LD-mitochondria contact sites (Miner et al. 2024). The lipid transfer protein VPS13D along with the TSG101 component of endoplasmic sorting complexes (ESCRT) required for transport, mediates FA trafficking from LDs to mitochondria through a specialized membrane scission mechanism (Wang et al. 2021b). Similarly, ABCD1, located on peroxisomal membranes, interacts with spastin (SPAST) on LDs, creating physical tethers (Henne 2019) and recruits ESCRT III proteins for FA trafficking from LDs to peroxisomes (Chang et al. 2019). The ARF1 GTPase also localizes to LD-mitochondria as well as LD-peroxisome contact sites and LD-mitochondria-peroxisome three-way contact sites, to regulate their formation and facilitate FA transfer (Chen et al. 2025; Kumar et al. 2024). Recent studies have also revealed that both LDs and peroxisomes form at the same specialized PEX30-enriched ER subdomains. The multiple C2 domain-containing transmembrane proteins (MCTPs) are considered to be mammalian homologues of the yeast protein, PEX30 (Joshi et al. 2018), and share a common molecular machinery required for LD and peroxisome budding from the ER (Wang et al. 2018) by coordinated membrane-remodeling mechanisms (Joshi and Cohen 2019), suggesting a link between the biogenesis of both organelles**.**

The FA-rich LD system in the RPE is essential for retinal function but can also become dysregulated with aging and stress, contributing to retinal degeneration (Han et al. 2024; Yako et al. 2022). The formation of LDs in the RPE has a physiological purpose in lipid management, where RPE serves as a lipid reservoir for the retina (Baker et al. 1986; SanGiovanni and Chew 2005). Beyond a passive role as storage sites, LDs in the RPE are dynamic organelles that participate in cellular metabolism and regulation. Considering that FA-fueled energy metabolism is a major energy source in the RPE, stimulating or increasing RPE FA β-oxidation leads to increased FA catabolism and decreased LD content (Hass et al. 2024). The presence of LDs also determines the metabolic state of the RPE, as droplet accumulation can be deleterious. Oleic acid treatment of ARPE-19 cells has shown increased LD formation leading to suppressed POS uptake; in turn, this uptake is restored by inhibiting LD formation (Yako et al. 2022). Thus, LDs play a dual role, on one hand preserving and chaperoning lipids like PUFAs for recycling and metabolism, and on the other hand serving a protective adaptation when POS-derived lipids temporarily exceed the RPE oxidative capacity. The composition of RPE LDs reflects this role. Pathological LD accumulation is observed in aging RPE and prominently in AMD, in association with oxidative stress, chronic inflammation, RPE dysregulation and secondary photoreceptor degeneration (Yako et al. 2022; Zhang et al. 2023). Additionally, multiple retinal mutations are also associated with LD accumulation (Gao et al. 2022; Hara et al. 2023; Ren et al. 2025). Overall, the interplay between LDs and PUFA in the RPE is central to normal retinal physiology and pathogenesis of retinal degeneration. The RPE has established mechanisms to form, store and mobilize PUFA-rich LDs to support retinal health. Yet, this system under stress and disease conditions might become locked into LD accumulation, fueling oxidative damage and inflammation. Understanding LDs-PUFA dynamics in the RPE might reveal novel therapeutic opportunities.

### Peroxisomes

#### Peroxisomal function and polyunsaturated fatty acids (PUFA) in the RPE

Peroxisomes are dynamic organelles whose numbers and size can change in response to proliferative and turnover signals, or cellular stressors (Smith and Aitchison 2013). Among their many cellular roles, peroxisomes in the RPE participate in lipid metabolism, harboring enzymes necessary for ꞵ-oxidation of very long chain FAs (VLCFA) and for a-oxidation of branch chained FAs (BCFA), which are components of POSs that are phagocytosed, degraded, and recycled by the RPE (Kevany and Palczewski 2010; Kocherlakota et al. 2023; Lodhi and Semenkovich 2014; Schrader and Fahimi 2008). POS-derived VLCFAs are initially shortened in RPE peroxisomes via ꞵ-oxidation, and the resultant shorter chain FAs are transferred to mitochondria for additional ꞵ-oxidation and energy production (Fliesler and Anderson 1983; Wanders et al. 2016). This catabolism of lipids (as well as that of amino acids and polyamines) generates hydrogen peroxide and reactive oxygen and nitrogen species (Fransen et al. 2012). Hydrogen peroxide is metabolized into water and oxygen by catalases found in six-fold higher concentrations in RPE peroxisomes compared to other ocular tissues (Liles et al. 1991). At physiological levels, these peroxisome-derived reactive oxygen species (ROS) and reactive nitrogen species (RNS) act as signaling molecules that modulate inflammation, innate immune responses, and cell fate transitions during development; whereas higher levels lead to damage of macromolecules, triggering oxidative stress and subsequent tissue damage (Antonenkov et al. 2010; Di Cara et al. 2019; Fransen et al. 2012; Nordgren and Fransen 2014).

Peroxisomes also participate in the final steps of DHA synthesis and initiate the biosynthesis of ether-linked phospholipids; processes important for membrane structural integrity, photoreceptor function, and cell survival (Dean and Lodhi 2018; Jo et al. 2020). In the ER of the RPE, dietary ALA undergoes a series of desaturation and elongation reactions to form a 24-carbon VLCFA with six double bonds: tetracosahexaenoic acid. In the peroxisomes, two carbon atoms are removed via b-oxidation to form DHA, which can then be transferred to PRs (Ferdinandusse et al. 2001). Ether lipid synthesis begins in the peroxisome through synthesis of the precursor 1-alkyl-glycerol-3-phosphate (1-alkyl-G3P), which is then transported to the ER for further processing. Within the ER, PUFAs can be incorporated at the sn-2 position of the glycerol backbone of ether phospholipids (ePLs) (Dean and Lodhi 2018). These PUFA-ePLs are important for modulating cell membrane properties that affect lipid signaling (Lodhi and Semenkovich 2014) and membrane fluidity (Harayama and Shimizu 2020). They also play a role in the regulation of ferroptosis (Huang et al. 2025). The incorporation of highly unsaturated FAs into ether lipids sensitizes cells to ferroptosis; however, PUFA containing plasmalogens (vinyl-ether phospholipids) have also been hypothesized to act as antioxidants (Cui et al. 2021; Huang et al. 2025).

#### Peroxisomes and posterior segment diseases

Peroxisomal diseases (Table 2) are generally classified as Peroxisome Biogenesis Disorders (PBD) or Single Enzyme/Protein Deficiency Disorders (SEPD) (https://thegfpd.org/peroxisomal-disorders/). PBDs include Zellweger spectrum disorders (ZSD) and Rhizomelic Chondrodysplasia Punctata (RCDP), which are caused primarily by loss of peroxin function. Peroxins are proteins that carry out peroxisome assembly and import of matrix proteins, as well as peroxisome proliferation and fission (Jansen et al. 2021; Steinberg et al. 2006). Examples of SEPDs include ACOX1 and AMACR deficiencies, as well as X-linked adrenoleukodystrophy and adult Refsum disease. These disorders can affect multiple organ systems and manifest as impaired growth, facial dysmorphisms, sensory and neurological defects, renal and endocrine disruptions, hypotonia, skeletal defects, and/or delays in development. The organ systems affected are those that rely heavily on peroxisomal lipid metabolism and its detoxification (Bose et al. 2020; Steinberg et al. 2006; Wanders and Waterham 2006; Waterham et al. 2016)]. The severity of these disorders depends on the type of genetic mutation and its effects on the function of the encoded protein (He et al. 2021; Preuss et al. 2002) as well as the genetic background in which the mutation occurs (Simons and Nowaczyk 2012), and on interactions with environmental factors (Baldwin et al. 2010). Early onset diseases with rapid progression tend to be more severe, with affected individuals dying within a year of birth. The effects of the underlying mutations on the RPE and retina are therefore rarely studied in severe cases, whereas milder mutations, which show greater preservation of peroxisomal function, lead to later-onset disease with affected individuals surviving into adulthood and are more frequently associated with vision impairment (Bose et al. 2022; Das et al. 2021). Additionally, reductions in peroxisome function have been implicated in common age-related disorders such as diabetes, cancer, and neurodegenerative diseases including age-related macular degeneration (Cipolla and Lodhi 2017; Fransen et al. 2013; Landowski et al. 2024; Zalckvar and Schuldiner 2022).

**Table 2:** Peroxisomal proteins, function and associated disease and models

Gene	OMIM #	Function	Diseases	Human ocular phenotypes	Biochemical alterations human*/mouse^#^	Mouse models retinal/ocular phenotypes
Peroxisome assembly						
*PEX3*	603164	PEX3 forms a complex with PEX19 (and possibly PEX16) (Ghaedi et al. 2000), acting as a docking site and sorting site for peroxisomal membrane proteins (PMPs) (Fujiki et al. 2006; Jansen and van der Klei 2019); the cytosolic portion of PEX3 also interacts with membrane lipids (Pinto et al. 2009)	Peroxisome Biogenesis Disorder (PBD) 10A PDB10A (Zellweger, MIM:614882)	Ophthalmoplegia, nystagmus, cataracts, pallor of optic disc, visual impairment with hyperopia (Lee et al. 2020b)	*Reduced peroxisome number that leads to impairment of VLCFA b-oxidation and plasmalogen synthesis, accumulation of toxic intermediates of VLCFAs that can lead to oxidative stress and tissue damage (Bjorgo et al. 2017; Lee et al. 2020b)	*Pex3*^*tm1a(EUCOMM)Wtsi*^ (MGI:4431895) KO present abnormal eye size and morphology
*PEX16*	603360	Necessary for initiation of peroxisome biogenesis at the ER; recruitment and trafficking of peroxisomal proteins (Kim and Mullen 2013)	PBD8A (Zellweger, MIM: 614876), PBD8B (MIM: 614877)	Cataract, optic atrophy (Ebberink et al. 2010), abnormal retinal pigmentation (Wehbe et al. 2025)	*Reduced, enlarged peroxisomes, elevated plasma VLCFA, BCFA and bile acid intermediates (Ebberink et al. 2010)	*Pex16*^*tm1c(EUCOMM)Hmgu*^ (MGI:6438234) Retina not examined (Chen et al. 2024)
*PEX19*	600279	Docking site for peroxisomal membrane protein that assists in guiding peroxisomal membrane proteins to destination by complexing with PEX3 (Jansen and van der Klei 2019; Matsuzono et al. 1999)	PBD12A (Zellweger, MIM: 614886)	Cataracts, LCA/EOSRD or RP, OA, cystoid macular edema, absent ERG response, RPE disruption (Zellweger Spectrum disorders, including PEX19) (Yergeau et al. 2023)	*Abnormal VLCFA, aberrant plasmalogen biosynthesis, reduced peroxisomal α- and β-oxidation (Mohamed et al. 2010)	*Pex19*^*em1(IMPC)Bay*^ (MGI:7425905) KO shows increase in corneal thickness, abnormal posterior eye chamber depth, and microphthalmia (Wilson et al. 2026a)
matrix import						
*PEX1*	602136	PEX1 and PEX6 form a heterohexameric AAA-ATPase dislocase which translocates ubiquinated PEX5 from the peroxisomal membrane to allow it to recycle, thus maintaining the import of matrix proteins (Judy et al. 2022)	Heimler syndrome1 (HS1, MIM:234580), (PBD)1A (Zellweger, MIM:214100), PBD1B (MIM:601539)	Late onset retinal or macular degeneration (HS1), Early- onset RP, Pigmentary retinopathy, Usher-like syndrome, macular edema (Ratbi et al. 2016; Yergeau et al. 2023)	*Inefficient peroxisomal import of proteins necessary for FA β-oxidation, plasmalogen synthesis. Accumulation of VLCFA (Walter et al. 2001). Mutations in *PEX1* are the most common, accounting for 60% of peroxisomal defects in ZSS disorder (Ebberink et al. 2011) ^#^Accumulation of C26:0 lysophosphatidylcholine (Argyriou et al. 2019; Hiebler et al. 2014)	*Pex1*^*tm1.1Sjms*^ (MGI:5570186) PEX1-p.Gly844Asp, Early attenuation of cone response and morphology with later gradual decrease in rod function, bipolar degeneration, IS disruption, enlarged mitochondria (Argyriou et al. 2019; Hiebler et al. 2014) and progressive retinal degeneration and inflammation (Omri et al. 2025)
*PEX2*	170993	Peroxisomal E3 ubiquitin ligase peroxin 2, part of the retro-translocation channel. Necessary for the recycling of PEX5 (Platta et al. 2009). Also involved in pexophagy (Sargent et al. 2016)	PBD5A (Zellweger, MIM: 614866), PDB5B (MIM: 614867)	Retinitis Pigmentosa (Gootjes et al. 2004a; Shimozawa et al. 1999) Optic nerve atrophy (Gootjes et al. 2004a)	*Accumulation of phytanic acid and VLCFAs (Shimozawa et al. 1999)	*Pex2*^*tm1Plf*^ (MGI:2180128) No ocular phenotype reported
*PEX5*	600414	Cytosolic receptor for peroxisomal matrix proteins that bear peroxisomal targeting signal 1 (PTS1) and imports enzymes into peroxisomes (Francisco et al. 2013; Skowyra and Rapoport 2022; Wang and Subramani 2017)	PBD2A (Zellweger, MIM:214110), PBD2B (MIM:202370), rhizomelic chondrodysplasia punctata type 5 (RCDP5), (MIM:616716)	Atypical, milder biogenesis disorder and retinitis pigmentosa (Whelan et al. 2022)	*Accumulation of plasma phytanic acid, decreased erythrocyte C16:0 plasmalogen levels (Fallatah et al. 2021)	*Pex5*^*tm1Pec*^ (MGI:2384516) Crossed to Tg(Crx-cre)764Gla (Baes et al. 2002), *CRX-Pex5*^*−/−*^ mice show lipid accumulation, normal PR number and scotopic a-wave ERG, abnormal scotopic b-wave ERG (Swinkels et al. 2022)
*PEX6*	601498	PEX6 together with PEX1 act as a AAA-ATPase motor, extracts PEX5 from the peroxisomal membrane during ATP dependent extraction event (Pedrosa et al. 2018)	Heimler syndrome 2 (MIM: 616617) (Daich Varela et al. 2020; Gao et al. 2019), PBD4A (MIM: 614862), PBD4B (MIM: 614863)	RP, pigmentary macular dystrophy, macular cysts, Heimler syndrome, Usher-like syndromes with hearing loss (Raas-Rothschild et al. 2002; Smith et al. 2016)	*Defect in import of peroxisomal matrix proteins that may lead to altered peroxisome number; VLCFA accumulation, plasmalogen deficiency. Mutations in *PEX6* account for 15% of peroxisomal defects (Benson et al. 2021; Ebberink et al. 2011)	*Pex6*^*em1(IMPC)Bay*^ (MGI:6414534), *Pex6*^*eml(IMPC)Tcp*^ (MGI:6358627) KO: Homozygotes have microphthalmia, PEX6 localizes in PR cilia (Zaki et al. 2016), retinal phenotype not reported
*PEX7*	601757	Forms a complex with PEX5 to import cargo proteins with PTS2 binding motifs into peroxisomes, translocation across the peroxisomal membrane (Ramon and Bartel 2010)	PBD9B (MIM:614879), RCPD1 (MIM: 215100)	Retinitis pigmentosa (Braverman et al. 2002; van den Brink et al. 2003)	*^#^Plasmalogen deficiency, impaired phytanic acid accumulation (Braverman et al. 2010; Motley et al. 2002) *Deficiency of multiple peroxisomal enzymes in vitro studies) (Motley et al. 2002)	*Pex7*^*tm1Nbra*^ (MGI:4443126) Anterior segment lens defects and cataract and decreased FA levels, abnormal plasmalogen biosynthesis and BCFA oxidation, impaired PTS2 peroxisomal protein import (Braverman et al. 2010)
*PEX12*	601758	Together with PEX2 and PEX10 forms a membrane embedded ubiquitin ligase complex, functions as a retrotranslocation pathway for import of receptors (Feng et al. 2022). Also regulates PEX5 recycling (Okumoto et al. 2014)	PBD3A (Zellweger, MIM: 614859), PBD3B (MIM: 266510)	Abnormal ERG, early Retinitis Pigmentosa (Gootjes et al. 2004b), Retinopathy and corneal clouding (Zaki et al. 2021)	*Presence of non-functional peroxisomal ghosts (Chang et al. 1999; Santos et al. 1988)	*Pex12*^*tm1(KOMP)Vlcg*^ (MGI:4452940) No ocular phenotype reported
*PEX13*	601789	Serves as a docking factor for PEX5 which acts as an import receptor for the peroxisome targeting signal PTS1 (Gould et al. 1996)	PBD11A (Zellweger, MIM: 614883), PBD11B (MIM: 614885), Neonatal Adrenoleukodystrophy (NALD) (MIM: 9480815)	Visual decline (Shimozawa et al. 1998), cherry-red spot of the macula and diffuse white dots on fundoscopy, RPE abnormalities by OCT (Borgia et al. 2022)	*Variable elevation of phytanic acid and VLCFAs, reduced number of enlarged peroxisomes, abnormal distribution and dysfunction of mitochondria (Borgia et al. 2022)	*Pex13*^*tm1.1Crne*^ (MGI:2384515) Neonatal lethality, peroxisomal and mitochondrial morphology and physiology affected. No ocular phenotype reported (Maxwell et al. 2003)
*PEX26*	608666	Peroxisomal docking factor for PEX1/PEX6 heterodimer (Matsumoto et al. 2003a; Weller et al. 2005)	PBD7A (Zellweger-MIM: 614872), PDB7B (MIM: 614873), Heimler syndrome (MIM:234580, MIM:616617)	Deteriorating vision, Retinitis punctata albescens, retinal degeneration (Kim et al. 2021; Neuhaus et al. 2017)	*PBD7A: no catalase or thiolase import in fibroblasts, PBD7B: reduced import of catalase and thiolase, mildly elevated VLCFA (Matsumoto et al. 2003b)	*Pex26*^*tm1.1(KOMP)Vlcg*^ (MGI:5548637) Embryonic lethality (Dickinson et al. 2016)
Metabolic processes						
FAs oxidation						
*ABCD1*	300371	Transport of VLCFA and CoA derivatives into peroxisomes (van Roermund et al. 2008; Wiesinger et al. 2013) and coupled hydrolysis (Kawaguchi et al. 2021)	X-linked Adrenoleuko-dystrophy (MIM: 300100)	Cataracts, optic atrophy, ganglion cell degeneration, macular pigmentary changes (Traboulsi and Maumenee 1987)	*Impaired ß-oxidation, accumulation of VLCFAs (Wiesinger et al. 2013)	*Abcd1*^*tm1a(EUCOMM)Wtsi*^ (MGI:5782014) KO mice axonal degeneration, cataracts, retina not reported (Lu et al. 1997; Morato et al. 2013)
*ACOX1*	609751	ACOX1 catalyzes the first step of peroxisomal ꞵ-oxidation (Oaxaca-Castillo et al. 2007)	Mitchell syndrome (MIM: 618960), Peroxisomal acyl-CoA oxidase deficiency (MIM: 264470)	Impaired vision, optic atrophy, PR degeneration, RPE abnormalities, ERG abnormalities (Ferdinandusse et al. 2007; Filippi et al. 2024; Poll-The et al. 1988; Traboulsi and Maumenee 1987)	*^#^ Accumulation of toxic VLCFAs (Ferdinandusse et al. 2007; Hein et al. 2008)	*Acox1*^*em1(IMPC)Bay*^ (MGI:7425487) KO, Growth retardation, infertility, hepatosteatosis (Fan et al. 1996; Sheridan et al. 2011) Retinal phenotype not reported
*AMACR*	604489	Alpha-Methylacyl-CoA Racemase catalyzes the conversion of α-methyl-branched-chain fatty acyl-CoA esters from the (R)- to the (S)-stereoisomers. Necessary for peroxisomal β-oxidation (Ferdinandusse et al. 2000b; Schmitz et al. 1995)	Alpha-methylacyl-CoA racemase deficiency (MIM: 614307) Bile acid synthesis defect, congenital, 4 (MIM: 214950)	Retinitis pigmentosa, visual impairment (Ferdinandusse et al. 2000a; McLean et al. 2002; Smith et al. 2010)	*Elevation of α-branched FAs and accumulation of bile acid synthesis intermediates. Hepatitis with hepatocyte necrosis (Setchell et al. 2003). Increased serum pristanic acid and bile acid intermediates (Smith et al. 2010)	*Amacr*^*tm1Jkh*^ (MGI:3044171) KO: No ocular phenotype reported (Savolainen et al. 2004)
*EHHADH*	607037	Peroxisomal oxidation of FAs (Klootwijk et al. 2014)	Fanconi renotubular syndrome 3 (MIM:615605)	Flecked retina (Al-Hazzaa and Ozand 1997)	*^#^Diminished peroxisome number, perinuclear clustering of residual peroxisomes (Kan et al. 2024) *Impaired mitochondrial oxidative phosphorylation (Klootwijk et al. 2014)	*Ehhadh*^*tm1Jkr*^ (MGI:1857810) No ocular phenotype reported (Qi et al. 1999)
*HSD17B4* *(MFP2)*	601860	Hydratase and dehydrogenase steps of β-oxidation for straight and branched chain FA, bile acid intermediates, 17b-hydroxysteroid dehydrogenase activity (Ferdinandusse et al. 2006b; Moller et al. 1999)	D-bifunctional protein deficiency (MIM:261515), Perrault syndrome 1 (MIM: 233400)	Progressive vision loss (Ferdinandusse et al. 2006a), Retinitis Pigmentosa (McMillan et al. 2012)	* Failure of β-oxidation, accumulation of VLCFA and bile acid intermediates leading to cellular toxicity, enlarged or decreased peroxisomes (Ferdinandusse et al. 2006a) ^#^ Reduced retinal DHA, increased n-6 FA, lysosomal dysfunction (Das et al. 2021; Kocherlakota et al. 2023)	*Hsd17b4*^*tm1Baes*^ (MGI:2385822) KO: POS shortening, mild PR degeneration, RPE proliferation, aberrant hexagonal RPE cell morphology (Das et al. 2021, 2020; Kocherlakota et al. 2023)
*PHYH*	602026	Phytanoyl-CoA hydroxylase (PHYH) catalyzes the second step in phytanic acid α-oxidation (Foulon et al. 2003)	Refsum disease (MIM: 266500)	Nystagmus, night blindness, abnormal ERG Retinitis Pigmentosa (Jansen et al. 1997)	*Accumulation of phytanic acid (Foulon et al. 2003)	*Phyh*^*tm1Safe*^ (MGI:3822777) KO: Mice on a phytol enriched diet develop Refsum Disease relevant phenotypes, no ocular phenotype reported (Ferdinandusse et al. 2008)
Antioxidant system						
*EPHX2*	132811	Catalyzes hydrolysis of epoxides, especially those derived from PUFA, into diols (Gautheron and Jeru 2020)	Hyper-cholesterolemia (MIM:143890)	Elevated epoxygenated FAs and inhibition of epoxide hydrolase can inhibit TNF-α induced inflammation in retinal vessels (Capozzi et al. 2016)	*Modulating FA signaling through disruption of lipid homeostasis, altered oxidative stress response (Gautheron and Jeru 2020)	*Ephx2*^*tm1Gonz*^ (MGI:2149064) KO: Elevation of EETs and reduced DHETs, abnormal retinal vasculature morphology with reduced endothelial cell proliferation, and attenuated secondary retinal capillary plexus (Hu et al. 2014)
*SOD1*	147450	Antioxidant that metabolizes superoxide radicals to molecular oxygen and hydrogen peroxide to prevent tissue damage from oxidative stress (Islinger et al. 2009)	Amyotrophic lateral sclerosis 1 (ALS1) (MIM:105400), Spastic tetraplegia and axial hypotonia (MIM:618598)	No human retinal phenotype reported	*Accumulation of ROS and ER stress (Suthar and Lee 2023)	*Sod1*^*tm1Leb*^ (MGI:1857873) KO: Auditory and retinal abnormalities, elevated ROS in RGC layer, decreased RGC number, thinning of nerve fiber layer, pattern ERG is reduced (Yuki et al. 2011)
Ether Lipid Synthesis						
*GNPAT*	602744	Catalyzes the first step of ether phospholipid biosynthesis (Honsho and Fujiki 2017)	Rhizomelic chondrodysplasia punctata, type 2 (RCDP2) (MIM:222765)	Cataracts only (MIM: 222765) (Affected children die young)	*^#^Reduced levels of plasmalogens and ether lipids cause membrane defects and impaired signaling pathways (Dorninger et al. 2017; Sztriha et al. 2000)	*Gnpat*^*tm1Just*^ (MGI:2670461) KO mice: increased mortality, microphthalmia, cataracts, RPE abnormalities (Rodemer et al. 2003)

Earlier studies have shown that peroxisome function is critical for normal ocular health. In a study examining multi-systemic effects across patients diagnosed with ZSD, 88.6% of patients (n = 79) had vision impairment (Bose et al. 2020). In a comprehensive review of clinical findings, combining literature with chart review of cohorts of patients with ZSD, 81.1% of subjects with severe, intermediate or mild disease reported vision loss (Bose et al. 2022). Disease phenotypes can range from anterior segment defects such as corneal opacities, cataracts, glaucoma or optic atrophy to posterior segment defects such as retinal ganglion cell, PR, and RPE degeneration or pigmentary dystrophy (Das et al. 2021; Folz and Trobe 1991). While most mutations in peroxins lead to posterior segment defects (9 of 15 in Table 2), mutations in PEX3, PEX11 and PEX15 present only with anterior segment ocular defects, namely cataracts, while mutations in PEX1, PEX16 and PEX19 manifest both. Loss or reduction of peroxin or peroxisome-associated enzyme function can lead to the ocular pathologies summarized in Table 2. These pathologies arise from the disruption of lipid metabolism, leading to accumulation of VLCFAs, BCFAs, and toxic intermediates that trigger oxidative stress and macromolecule, cell, and tissue damage (Chen et al. 2022; Morito et al. 2024; Yu et al. 2022). Alternately, impaired biosynthesis of molecules such as DHA and ether-lipid phospholipids necessary for membrane structure, integrity, and signaling (Jo et al. 2020) can also lead to ocular pathologies.

### Mitochondria

#### Mitochondrial function and polyunsaturated fatty acids (PUFA) in the RPE

Mitochondrial function in the RPE is crucial for PUFA metabolism. RPE mitochondria are uniquely adapted to manage high oxidative stress, intense phagocytic activity and lipid metabolism to support the overlying PR cells (Brown et al. 2019; Markitantova and Simirskii 2025; Tong et al. 2022). As a result, the RPE relies heavily on mitochondria for its high energy requirements and preferentially uses FAs to produce ATP to perform its many functions (Hansman et al. 2025; Nolan et al. 2021). The major FAs utilized in the RPE are LC-PUFAs such as DHA and VLC-PUFAs, which are obtained through the phagocytosis of POS (Kevany and Palczewski 2010; Nolan et al. 2021; Nwagbo and Bernstein 2023). Both peroxisomes and mitochondria work in close metabolic coordination in the RPE, where peroxisomes initiate cleavage of LC- and VLC-PUFAs to form shorter chain FAs. The process is continued in the mitochondria through β-oxidation to produce ATP or generate ketone bodies, which are then transported back to PR to be used as an alternate fuel source (Adijanto et al. 2014).

For mitochondrial FA metabolism, fatty acyl CoA derivatives are transported from the cytosol into the mitochondrial matrix by the carnitine shuttle (CPT1, CACT and CPT2) (Dikalov et al. 2024). Once inside the mitochondrial matrix, sequential actions of the enzymes of the β-oxidation pathway—acyl-CoA dehydrogenases (ACADs such as VLCAD, MCAD, SCAD, LCAD) (He et al. 2011), ECHS1 (Fu et al. 2025), HADHA/HADHB (Mitochondrial Trifunctional Protein; MTP) (Diebold et al. 2019) and ACAA2 perform the repetitive cleavage steps that result in acetyl-CoA production (Zhang et al. 2019). Electrons from the oxidation reactions are passed via Electron-Transferring Flavoprotein (ETF) and ETC Dehydrogenase (ETFDH) to the mitochondrial electron transport chain (ETC), coupling FA catabolism to oxidative phosphorylation and energy (ATP) generation (Wang et al. 2019).​ The acetyl-CoA then either enters the TCA cycle or is shunted into the ketogenic pathway to produce β-hydroxybutyrate to be used by PR cells for energy (Adijanto et al. 2014).

#### Mitochondrial disorders affect PUFA metabolism in the RPE

Mitochondrial disorders that affect FA metabolism (including PUFA), are broadly grouped into FA oxidation disorders (FAODs). FAODs are a group of inherited metabolic diseases caused by disruption in one of the genes involved in either mitochondrial β-oxidation or FA transport via the carnitine cycle (Merritt et al. 2018) (Table 3). Like other mitochondrial disorders, symptoms of FAODs can manifest in different tissues that rely on energy production via FA oxidation and appear as neurological defects including developmental delays and peripheral neuropathy, hepatic defects including hypoglycemia and hepatic dysfunction, skeletal myopathy and cardiomyopathy (Merritt et al. 2020). These defects also affect metabolically demanding eye tissues and may appear as retinopathies with damage to the retina and symptoms including progressive retinal dysfunction and vision loss. The underlying cause is attributed to the inability of the mitochondria to break down FAs, which can accumulate, forming toxic intermediates that harm the RPE and PR cells (Fu et al. 2021; Joyal et al. 2016).

**Table 3:** Mitochondrial proteins, function and associated disease and models

Gene	OMIM #	Function	Diseases	Human Ocular phenotypes	Biochemical alterations in human*/mice^#^	Mouse Models Retinal/Ocular Phenotypes
FA β-oxidation						
*DECR1*	222745	Auxiliary enzyme that catalyzes the rate limiting step in PUFA oxidation, converting 2,4-dienoyl-CoA into 3-trans-enoyl-CoA which then enters the standard β-oxidation pathway	DECR1 deficiency (MIM:616034)	Childhood-onset optic atrophy (in milder cases) (Pomerantz et al. 2018) cortical blindness (in severe encephalopathic cases), nystagmus (as part of neurological involvement) (Houten et al. 2014)	*Hepatic form – hepatomegaly, elevated serum transaminases, steatotic liver disease (Kohlmaier et al. 2024), Secondary DECR1 deficiency due to mutation in NADK2 results in failure to thrive, hypertonia, choreoathetosis and dystonia, epilepsy pancreatitis, progressive leukodystrophy, cerebral atrophy, death at childhood (Houten et al. 2014)	*Decr1*^*tm1Jkh*^ (MGI:4355415) No ocular phenotype reported (Miinalainen et al. 2009)
*ACAD9*	611103	Two main functions – break down of fats (oxidation) and assembly of complex I (energy production)	Mitochondrial Complex I Deficiency (MIM: 611126)	Optic neuropathy (Gueguen et al. 2021)	*Affects brain, liver, heart, kidney and nerves with accumulation of lactic acid. weakened heart muscle, which is typically fatal in infancy or childhood (Haack et al. 2010)	*Acad9*^*tm1a(KOMP)Wtsi*^ (MGI:4944332) Homozygous KO mice appear to be lethal Conditional mice available *Acad9*^*tm1c(KOMP)Wtsi*^ (MGI:7327433) Cardiac-specific deficiency results in neonatal cardiomyopathy and mice die by 17 days of age. No ocular phenotype reported (Sinsheimer et al. 2021)
*ECHS1*	602292	Encodes short-chain enoyl-CoA hydratase, which functions in the second step of the FA oxidation and hydrates 2-trans-enoyl-coenzyme A intermediates to L-3-hydroxyacyl-CoAs	ECHS1 deficiency (MIM:616277)	Optic atrophy and nystagmus (Bernhardt et al. 2024)	*Metabolic encephalopathy (Masnada et al. 2020)	*Echs1*^*em1Lutzy*^ (MGI:8282681) p.F33S homozygotes have normal appearance and survival *Echs1*^*em3Lutzy*^ (MGI:8282682) Homozygous KO mice show prenatal lethality (Eller et al. 2024)
*HADHA*	600890	α-subunit of the trifunctional protein, a complex enzyme crucial for breaking down long-chain FAs	LCHAD deficiency (MIM: 609016), Mitochondrial trifunctional protein deficiency 1 (MTPD1) (MIM: 609015)	Choroidal atrophy, disorganization of the outer retinal layer, myopia (Lange et al. 2024)	*Higher total body fat and extramyocellular lipid deposition, higher plasma long-chain acylcarnitine and long-chain hydroxyacylcarnitine levels (Gillingham et al. 2013; Van Hove et al. 2000)	*Hadha*^*em1Mbg*^ (MGI:7266257) Preweaning lethality, incomplete penetrance, RPE degeneration, 53% eyes have hypopigmented areas, decreased photopic b- and c-wave amplitudes. Accumulates plasma 3-hydroxyacylcarnitine, decreased FA oxidation in heart and liver (Gaston et al. 2023)
*HADHB*	143450	Provides instructions for making the β-subunit of the mitochondrial trifunctional protein, contains 3-ketoacyl-CoA thiolase activity	Mitochondrial trifunctional protein deficiency 2 (MTPD2) (MIM: 620300)	Retinopathy (Fletcher et al. 2012)	*Multi-organ involvement; severe neonatal form with cardiomyopathy, hepatic form with recurrent hypoketotic hypoglycemia, and later-onset axonal sensory neuropathy with episodic myoglobinuria (Orstavik et al. 2022). ^#^Premature sudden death between 9 and 16 months of age, cardiac fibrosis, cardiomyopathy with increased fat accumulation, abnormal brown adipose tissue morphology, hepatic steatosis, elevation of long chain acylcarnitine levels. (Kao et al. 2006)	*Hadhb*^*m1Ytc*^ (MGI:3698125) No ocular phenotype reported (Kao et al. 2006)
Mitochondrial dynamics						
*MFN2*	608507	During mitochondrial fusion, regulates fusion of the outer mitochondrial membranes	Charcot-Marie-Tooth (CMT) disease type 2A (MIM: 609260, 617087), Multiple Symmetrical Lipomatosis (MSL) (MIM: 151800)	Optic atrophy (Guerriero et al. 2020), Cataracts (Nan et al. 2021)	*MSL – lipodystrophy and lipomatosis, lipoatrophy, insulin resistance and/or diabetes. CMT – mitochondrial alterations, decreased leptin and adiponectin expression (Capel et al. 2018) ^#^*Mfn2*^*R707W*^ knock-in mice show adipose-specific mitochondrial morphological abnormalities and reduced secretion of leptin and adiponectin in serum (Mann et al. 2023)	*Mfn2*^*tm1Dcc*^ (MGI:2450306) Homozygous KO mice show embryonic lethality (Chen et al. 2003) *Mfn2*^*tm3Dcc*^ (MGI:3779081) Conditional KO induces cataracts in mice (Zhao et al. 2018)
*OPA1*	605290	Dynamin related GTPase. During mitochondrial fusion, regulates fusion of the inner mitochondrial membranes	Autosomal Dominant Optic Atrophy (ADOA) (MIM: 165500), Behr Syndrome (MIM: 210000), Mitochondrial DNA depletion syndrome 14A (MIM: 621481)	Progressive bilateral optic atrophy, central visual field and color vision defects (Lenaers et al. 2012; Othman et al. 2022) Reduced retinal nerve fiber and ganglion cell layer thickness (Schild et al. 2013)	*Impaired ATP synthesis through complex I (Carelli et al. 2015) ^#^*Opa1*^±^ mice show a 46% decrease in complex IV cytochrome oxidase activity. *Opa1*^*delTTAG*^ mice have multi-systemic poly-degenerative phenotype, with decreased cytochrome oxidase staining in the retina (Sarzi et al. 2012)	*Opa1*^*M1Bewi*^ (MGI:4412032) Homozygotes are embryonic lethal, heterozygous mice have decreased RGC numbers, abnormal optic nerve morphology, thin IPL (Alavi et al. 2007) *Opa1*^*tm1.1Geno*^ (MGI:6117138) loss of RGC, optic nerve defects, progressive vision failure, altered mitochondrial numbers (Sarzi et al. 2012)
*DNM1L*	603850	Mediates mitochondrial fission	DNM1L-Related Encephalopathy (MIM: 614388), Optic atrophy 5 (MIM: 610708)	Optic atrophy and vision loss (Gerber et al. 2017)	*Severe early-onset form – epileptic encephalopathy, severe developmental delay, early death and profound mitochondrial dysfunction, Intermediate form – sudden onset refractory status epilepticus, epileptic encephalopathy, Milder form – isolated optic neuropathy, paraparesis Elongated and hyperfused mitochondria, and functions associated with mitochondria including energy/calcium/FA metabolism are altered (Liu et al. 2021; Manting et al. 2025)	*Dnm1l*^*tm1b(KOMP)Wtsi*^ (MGI:5602830) Homozygotes are embryonic lethal. Heterozygotes have abnormal optic disc, cornea and vitreous body morphology (IMPC (Wilson et al. 2026b))
*TMEM135*	616360	Suggested to regulate mitochondrial fission by activating DRP1 (Landowski et al. 2024)	No mendelian human disease known	No human eye phenotype reported	*No causative mutations but associations of genetic variants (rs567403 C > G) with cutaneous melanoma-specific survival (Wang et al. 2021a)	*Tmem135*^*fun025*^ (MGI:5811600) Loss of function leads to photoreceptor degeneration, RPE hyperplasia, abnormal rod and cone electrophysiology, abnormal mitochondrial morphology with decreased numbers and increased size, abnormal ETC with decreased maximal respiration and spare respiratory capacity, decreased mitochondrial fission, accumulation of auto-fluorescent cells and aggregates between photoreceptors and RPE, lipid accumulation (Landowski et al. 2020; Lee et al. 2016)
Mitochondrial regulation						
*PPARG*	601487	Ligand-activated transcription factor (Zhang et al. 2015), involved in lipid metabolism (Ershov and Bazan 2000)	Familial Partial Lipodystrophy Type 3 (FPLD3) (MIM: 604367)	No direct ocular defects. FPLD3 patients who develop diabetes may develop diabetic retinopathy (Wu et al. 2024)	*Early onset Type 2 diabetes mellitus, atherogenic dyslipidemia (high TGs, low HDL), arterial hypertension, cardiovascular disease, fatty liver, fat distribution abnormalities with loss of subcutaneous fat from extremities (Akinci et al. 2017; Soares et al. 2024)	*Pparg*^*tm1Avp*^ (MGI:3694813) Homozygotes show preweaning lethality *Pparg*^*tm1Tka*^ (MGI:2429766) Following treatment with STZ, heterozygous mutant mice exhibit a 2.1-fold increase in retinal leukostasis and a 1.9-fold increase in retinal vascular leakage (Muranaka et al. 2006)
*PPARA*	170998	Transcription factor, regulator of FA metabolism—PUFAs can bind to PPARα, causing it to activate transcription of genes responsible for ß-oxidation	No monogenic disease phenotypes known	No ocular defects reported	*Modifier gene for Familial Combined Hyperlipidemia (FCHL) (Eurlings et al. 2002) ^#^Impaired use of lipids for oxidative phosphorylation in *Ppara*^*−/−*^ mouse retina (Pearsall et al. 2017)	*Ppara*^*tm1Gonz*^ (MGI:1857774) Mice develop retinal degeneration at 8 weeks of age, retinal bioenergetics deficiency and neurodegeneration (Pearsall et al. 2017)
*PPARGC1A*	604,517	Master regulator of mitochondrial function; coactivator of nuclear receptors and other transcriptional factors that regulate mt. biogenesis; transcriptional control of nuclear genes encoding mtFAO enzymes (Vega et al. 2000)	No monogenic disease phenotypes known	No ocular defects reported	*Reduced expression of *PPARGC1A* in iPSC-RPE cells from AMD donor RPE compared to control cells (Golestaneh et al. 2016) *PPARGC1A* knockdown in ARPE-19 cells showed reduced oxidative phosphorylation capacity (Rosales et al. 2019), and LD accumulation in ARPE19-PGC1A KO cells (Zhou et al. 2024)	*Ppargc1a*^*tm1Brsp*^ (MGI:3511352) Homozygous null mice display partial postnatal lethality (Lin et al. 2004) Heterozygotes fed a high fat diet at 3 months and then observed 4 months later (at 7 months) showed abnormal choriocapillaris morphology, short PR inner and outer segments, PR and RPE degeneration, accumulation of lipofuscin in the cytoplasm of RPE cells, basal laminar deposits, and thickening of outer collagenous layer, decreased mt. activity and autophagy dynamics/flux, increased ROS, and inflammatory response in RPE and retina (Zhang et al. 2018)
Subunits of mitochondrial ETC chain and oxidative phosphorylation						
*MT-ND* genes	*ND1*-516000; *ND2*- 516001; *ND3*-516002; *ND4*-516003; *ND4L*- 516004; *ND5*- 516005; *ND6* 516006	Genes encode subunits of the Complex I of the mitochondrial ETC chain	Leber’s Hereditary Optic Neuropathy (LHON) (MIM:535000), mitochondrial encephalo-myopathy, lactic acidosis, and stroke-like episodes (MELAS) (MIM:540000), Leigh syndrome (MIM:500017)	LHON – sudden, painless central vision loss in young adults (Berardo et al. 2020) MELAS – optic neuropathy (Scarcella et al. 2023)	*Multisystemic abnormalities, decreased mitochondrial protein levels and respiration (Ren et al. 2022), lactic acidosis (Spruijt et al. 2007), decreased mitochondrial respiratory chain complex I activity (Lin et al. 2014), decreased mitochondrial membrane potential and increased ROS (Lou et al. 2023)	Del(MTmt-Tk-mt-Nd5)1Jiha (MGI:3776665) Heteroplasmic *mt-Nd5* knockout mice present damaged mt. cristae in the cerebral cortex, hippocampal atrophy and asymmetry, resulting in learning and memory abnormalities. Mice also susceptible to obesity and thermogenic disorders (Kim et al. 2024) Allotropic expression of the mutant human ND4 in the mouse visual system induced symptoms of LHON, including swelling of the optic nerve head, disrupted mt. cytoarchitecture, elevated ROS, apoptosis and progressive ganglion cell death (Qi et al. 2007)
*NDUFS4*	602694	Nuclear genes encode subunit of the Complex I of the mitochondrial ETC chain	Mitochondrial Complex I deficiency Nuclear Type 1; MC1DN1 (MIM:252010), Leigh Syndrome (MIM:256000)	Ocular atrophy, nystagmus (Koene et al. 2012)	*Heterogeneity and multi-organ involvement in phenotypes – elevated lactate levels, defects in mitochondrial complex I assembly (Scacco et al. 2003; Shamriz et al. 2025)	*Ndufs4*^*tm1.1Rpa*^ (MGI:3793713) Homozygous mice die around 7 weeks of age. Mice lack complex I activity. Eyes develop cataracts and blindness by P20 (Kruse et al. 2008)
*MT-ATP6*	516060	Component of the ATP synthase enzyme, a protein complex that is essential for ATP generation	Neuropathy, Ataxia and Retinitis Pigmentosa (NARP) (MIM: 551500), Leigh syndrome (MIM:256000)	Retinitis Pigmentosa, ocular atrophy (Choi et al. 2024)	*Reduced basal respiration, increased ROS, abnormal mitochondrial cristae morphology (Ganetzky et al. 2019; Stendel et al. 2020) ^#^Mouse model shows decreased ATP synthesis and impaired oxidative phosphorylation in retina (Yuan et al. 2022)	Tg(ATP6*)#aJguy (MGI:8286375) Transgenic mouse model expressing a human ATP6 gene with a T > G mutation at position 8993 exhibits early death, paralysis, hunching, vision loss and seizures (Yuan et al. 2022)
Additional						
*SOD2*	147460	Neutralizes superoxide radicals (Flynn and Melov 2013)	Associated with neurodegeneration, premature aging, heart diseases (Flynn and Melov 2013)	No ocular phenotype identified	^#^Increased oxidation of DHA containing lipids, elevated levels of A2E and iso-A2E (Justilien et al. 2007)	*Sod2*^*tm1Cje*^ (MGI:1857344) The homozygous KO allele leads to perinatal lethality. 9–10 days old mice show thinning of photoreceptor layer and RPE, mitochondrial morphological abnormalities (Sandbach et al. 2001) *Sod2*^*tm1Kskk*^ (MGI:5774733) Conditional loss of SOD2 in RPE results in increased oxidative stress and autofluorescence in the RPE, reduced scotopic ERG response and thinning of PR layers and fundus abnormalities with leaky vessels (Mao et al. 2014)
*POLG*	174763	Mitochondrial DNA replication and repair	Mitochondrial DNA depletion syndrome 4A (Alpers type) (MIM: 203700, 613662), mitochondrial recessive ataxia syndrome (MMIM:607459), progressive external ophthalmoplegia (PEO) (MIM:157640, 258450)	PEO, cataracts and significant lens opacities (Rahman and Copeland 2019; Van Goethem et al. 2001)	*Mitochondrial dysfunction due to multiple deletions, oxidative phosphorylation defects (de Vries et al. 2007; Wong et al. 2008)	*Polg*^*tm1Prol*^ (MGI:3583920) Mice die of severe anemia by 15 months of age, effect on hematopoetic system, abnormal mitochondrial physiology. No eye phenotype reported (Chen et al. 2009)
*TFAM*	600438	Transcription and packaging factors for mitochondrial DNA; essential for regulating copy number and stability of mitochondrial genome	Mitochondrial DNA depletion syndrome 15 (hepatocerebral type) (MIM: 617156)	Ocular surface damage (Li et al. 2024)	*Neonatal liver failure, hypoglycemia (Stiles et al. 2016), impaired mitochondrial transcription initiation, reduced mitochondrial copy numbers and defective nucleoid formation (Mehmedovic et al. 2022), increased risk of developing neurodegenerative diseases (Song et al. 2024)	*Tfam*^*tm1.1Lrsn*^ (MGI:1860956) Embryonic lethality, Heterozygous mice have optic disc absence, abnormal heart and decreased body size, decreased mitochondrial DNA content with disorganized cristae and increased mitochondrial size and increased apoptosis and impaired OXPHOS (Larsson et al. 1998)

Mitochondrial associated disorders due to mutations in non-FA associated mitochondrial or nuclear-encoded genes have also been reported to result in defects affecting vision, such as Leber’s Hereditary Optic Neuropathy (LHON) due to mutations in mitochondrially-encoded genes such as *MT-ND1*, *MT-ND4* and *MT-ND6* (Manickam et al. 2017), Chronic Progressive External Ophthalmoplegia (CPEO) due to large-scale deletions in mitochondrial DNA or nuclear genes such as *POLG* or *TYMP* (Ali et al. 2024), Kearns-Sayre Syndrome (KSS) due to large-scale deletions in the mitochondrial DNA (Padhy et al. 2018; Zhu et al. 2022), MELAS due to mutations in *MT-TL1* (Keilland et al. 2016), NARP due to mutations in the mitochondrial gene *MT-ATP6* (Blanco-Grau et al. 2013), and Autosomal Dominant Optic Atrophy (ADOA) due to mutations in the nuclear-encoded *OPA1* gene (Ranieri et al. 2012). These disorders can cause a range of vision problems, including optic atrophy, retinopathy, and ophthalmoplegia (paralysis of the eye muscles). Importantly, many mitochondrial diseases are embryonic lethal or present severe phenotypes in other tissues like heart or liver. In such instances, ocular phenotypes have not been documented. Mouse models for mitochondrial mutations have been useful in understanding the disease pathologies and progression and are mentioned in Table 3 whenever available and studied.

Interestingly, the mitochondrial β-oxidation pathway has also been implicated in the synthesis of DHA from ALA, thereby suggesting the role of RPE mitochondria in PUFA synthesis, in addition to its breakdown (Harding et al. 1999). In addition to the primary mitochondrial diseases that result from direct impairment of mitochondrial functions, several ophthalmologic diseases which are not traditionally considered to have obvious mitochondrial origins are now being recognized to result in part from secondary mitochondrial dysfunctions that result either from environmental factors and/or other genetic disorders such as increased oxidative stress and apoptosis (Schrier and Falk 2011). Examples of such diseases include diabetic retinopathy, age-related macular degeneration and glaucoma (Eells 2019; Sun et al. 2025).

## PUFA efflux from the RPE to the PR cell

The final process required for retinal lipid homeostasis is the efflux of PUFAs from the RPE cell and their delivery to the retina. Classic tracer work in frog eyes provided direct evidence that the RPE participates in ocular DHA conservation. Movement of labeled DHA from phagocytosed POSs back to photoreceptors (Gordon and Bazan 1990; Gordon et al. 1992; Rodriguez de Turco et al. 1999) and in vitro biochemical studies demonstrated selective handling of DHA after outer segment phagocytosis and efficient return of DHA to PRs (Rodriguez de Turco et al. 1999). Around 80% of the DHA phagocytosed by the RPE is estimated to be recycled to PRs for new membrane synthesis (Chen and Anderson 1993; Storm et al. 2020). However, the underlying players and mechanisms that enable PUFA recycling remain unknown. Below, we consider multiple transporters that may be involved in transporting PUFAs from the RPE to the retina.

One of the key players for efflux from the RPE to retina is the interphotoreceptor retinoid binding protein (IRBP), also known as retinol binding protein 3 (RBP3) (Zeng et al. 2020). It shuttles 11-cis retinal and all-trans retinol between photoreceptor and RPE (Cunningham and Gonzalez-Fernandez 2003; Parker et al. 2011), but has also been shown to bind LCFAs like DHA (Semenova and Converse 2003). DHA can occupy a hydrophobic binding pocket and modulate IRBPs binding to retinoids (Chen et al. 1996, 1993), indicating that IRBP can carry FAs. IRBP might serve as an extracellular carrier that picks up DHA released from the RPE and transporting it to PRs.

The RPE apical membrane also contains the ATP‑binding cassette transporter A1 (ABCA1), an active transporter that serves as a key efflux route by promoting the loading of cholesterol and lipids onto APOA1 and HDL‑like particles. ABCA1 and ABCG1 act coordinately to support HDL biogenesis and maturation (Vedhachalam et al. 2007). In RPE-specific *Abca1/Abcg1* knockout mice, lipidomic analysis of eyecups revealed a marked accumulation of cholesteryl esters, including species esterified with palmitate, oleate and DHA, consistent with impaired cholesterol/phospholipid efflux and compensatory esterification/storage within the RPE (Storti et al. 2019). These findings align with polarized human RPE studies demonstrating directional ABCA1-dependent efflux from both apical and basal surfaces (Lyssenko et al. 2018; Storti et al. 2017). Although ABCA1-mediated export could in principle contribute to delivery of PUFA from the RPE to PRs, direct in vivo evidence demonstrating this transport remains limited.

The RPE can also secrete lipoprotein-like particles, including APOE and MTTP/APOB-dependent ApoB lipoprotein particles, which carry cholesteryl esters and TGs (Grubaugh et al. 2024; Ishida et al. 2004). These secreted particles may be plausible vehicles for transporting PUFA in esterified and/or TG form toward the subretinal space/PRs and would be more compatible with the finding that DHA is primarily incorporated into TGs in the RPE (Chen and Anderson 1993). However, the extent to which lipoprotein-like particles contribute to physiological PUFA supply to PRs remains to be studied.

Additionally, small extracellular vehicles, exosomes, could play a role in FA transport. RPE cells under stress have been shown to release exosomes containing lipids and protein waste, which might contribute to the transport between RPE and PR (Storm et al. 2020). While multiple mechanisms for PUFA transport between RPE and PR have been proposed, these pathways are likely to operate in parallel. Further studies are needed to delineate their relative contribution to PUFA efflux from the RPE.

## Summary and future directions

In this review, we highlighted the central role of the RPE in regulating the uptake, intracellular processing, retention and distribution of PUFAs, functions that are essential for PR development, maintenance, and long-term function. We summarized the molecular pathways governing PUFA influx, metabolic remodeling, storage and export within an organelle specific framework, using genetic loss-of-function phenotypes, where available, to illuminate molecular functions. Several RPE intrinsic defects provide strong evidence that disrupted PUFA metabolism within the RPE is sufficient to drive retinal disease, which collectively lead to PUFA imbalance, lipid accumulation, immune cell infiltration and progressive degeneration. In contrast, for many PUFA-related pathways that are shared across retinal cell types, additional experiments are required to resolve the relative contributions of the RPE versus those of the neural retina.

Importantly, retinal degeneration directly associated with disrupted PUFA homeostasis in the RPE may arise through mechanistically distinct pathways with distinct therapeutic implications. In some contexts, disease reflects insufficient PUFA availability to PRs, such as impaired DHA uptake or recycling, resulting in defective POS renewal. In others, pathology is driven by the accumulation of toxic PUFA intermediates or incompletely metabolized LC and VLC-PUFAs within the RPE, leading to cellular stress, including ER stress, oxidative stress and subsequently cell death. These divergent mechanisms imply different intervention strategies. Whereas PUFA deficiency may require restoration of lipid supply or recycling, disease driven by lipid overload may benefit from strategies that promote lipid clearance.

Significant gaps remain in our understanding of how PUFA pathways in the RPE support retinal homeostasis. These gaps are compounded by limited tissue accessibility, phenotypic complexity across disease models, and a lack of RPE-specific experimental models suitable for dissecting shared metabolic pathways and organelle functions. Addressing these challenges will require integrated approaches, including the development of improved disease models, and multi-omic (genomic, proteomic, and lipidomic) platforms, particularly more accessible and cost-effective lipidomic tools comparable in ease and affordability to current genomic and proteomic methods. A deeper understanding on how aging, environmental stress and epigenetic regulators shape RPE lipid metabolism may ultimately reveal new therapeutic strategies that preserve PUFA homeostasis, sustain POS renewal and slow degeneration in the diseased eye.

## Data Availability

No datasets were generated or analysed during the current study.
